# Before thalidomide: Heinrich Mückter and the Nazi typhus complex

**DOI:** 10.1017/mdh.2026.10059

**Published:** 2026-07

**Authors:** Lukas Frank, Dominik Groß, Nico Biermanns

**Affiliations:** Institute for History, Theory and Ethics of Medicine, Medical Faculty, RWTH Aachen University, Aachen, Germany

**Keywords:** Contergan, Delousing, Vaccine research, Human experimentation, Buchenwald, Holocaust

## Abstract

The physician and chemist Heinrich Mückter (1914–87) is widely known for his role in developing thalidomide at Grünenthal, whose market launch led to one of the most serious pharmaceutical scandals in history. Less scholarly attention has been paid, however, to his involvement in Nazi Germany’s typhus control and vaccine research and production between 1942 and 1945. Drawing on a variety of historical sources, this article reconstructs his work at a delousing facility in the Białystok District and at the Institute for Typhus and Virus Research of the German Army High Command in Krakow under hygienist Hermann Eyer (1906–97). We show that Mückter participated in a racially framed typhus control programme and in ethically dubious vaccine production methods. He also conducted unethical and methodologically questionable vaccine experiments on human beings in Krakow, including underage test subjects and exploiting the structural vulnerability of the occupied Polish population. In addition, we reassess assumptions about cooperation between Eyer’s Wehrmacht institute and the research station at Buchenwald concentration camp under SS physician Erwin Ding-Schuler (1912–45). Taken together, we argue that Mückter operated within a broader network of civilian and military scientists, health officials, and representatives of the pharmaceutical industry who accelerated the erosion of ethical boundaries in biomedical research and contributed to an exterminatory health and population policy in the occupied East. These practices, termed the ‘Nazi typhus complex’, reveal how preventive medicine, biomedical research, and exterminatory policies were intertwined in the context of an anti-Slavic war of annihilation and the Holocaust.

## Introduction

The name of the German physician and chemist Heinrich Mückter (1914–87) is commonly associated with the thalidomide scandal, one of the most serious pharmaceutical scandals in history. The active ingredient thalidomide (alpha-phthalimido-glutarimide) was synthesised in the spring of 1954 by two newly hired members of the research staff at the pharmaceutical company Chemie Grünenthal in Stolberg near Aachen: pharmacologist Herbert Keller (1925–2001) and pharmacist Wilhelm Kunz (b. 1920). It was first marketed as the combination drug *Grippex* against common colds in late 1956, and in 1957 as the sedative *Contergan.* Use of the drug during pregnancy caused damage to embryonic development, resulting in severe congenital malformations. As head of Grünenthal’s research department, Heinrich Mückter must be regarded as a key figure in the development, testing, and market launch of the drug.^1^

To this day, vague, oversimplified, and inaccurate accounts circulate regarding Heinrich Mückter’s activities during the Second World War (1939–45). In the leading German news magazine *Der Spiegel*, journalist Armin D. Steuer referred to Mückter’s Nazi past. According to Steuer, in the post-war period Mückter accepted the harm caused by thalidomide just as he had previously accepted harm to the health of concentration camp inmates in Buchenwald – ‘possibly also in Auschwitz’ – through vaccine experiments.^2^ Some thalidomide survivors have even promoted the dubious conspiracy theory^3^ that thalidomide was synthesised as early as 1944 as an antidote to the nerve agent sarin (designated ‘drug #4589’)^4^ and that it was ‘tested on women prisoners of war at the Auschwitz concentration camp by Doctors Heinrich Mückter and Otto Ambros,^5^ who worked under the supervision of Mengele’.^6^ However, drawing on Steuer’s reference to the vaccine trials at Buchenwald, some scholarly publications have also linked Mückter to human experiments on concentration camp inmates, in which typhus vaccine from the Institute for Typhus and Virus Research (*Institut für Fleckfieber- und Virusforschung*) of the Army High Command (*Oberkommando des Heeres*, OKH) in Krakow was tested.^7^ Anne Crumbach even states that Mückter himself carried out experiments on concentration camp inmates.^8^ Gine Elsner merely notes that Mückter had been accused by the Polish judiciary of involvement in vaccine experiments.^9^ Ernst Klee, by contrast, refers only to Mückter’s affiliation with the Institute for Typhus and Virus Research of the OKH.^10^

The typhus experiments conducted at the Buchenwald concentration camp near Weimar were mentioned by the former inmate and medical clerk Eugen Kogon (1903–87) in his influential work *Der SS-Staat: Das System der deutschen Konzentrationslager* [The SS State: The System of German Concentration Camps].^11^ They were part of the German efforts to develop an effective typhus vaccine for use by German troops on the Eastern Front. With Hitler’s invasion of Poland in September 1939, the diagnosis, treatment, prevention, and study of typhus became a priority for German physicians, health officials, and scientists.^12^ The Germans undertook a range of measures to counter the threat of typhus, pursuing two main strategies: active immunisation – primarily through vaccines – and the eradication of lice infestations through physical and chemical means.^13^ The segregation and ghettoisation of the Jewish population in the occupied eastern territories must also be seen in this context, as the racist stereotype of typhus as a distinctly ‘Jewish disease’ played a significant role.^14^

The history of Nazi typhus control and research is comparatively well researched, as is the disease’s significance for Nazi health and population policy, particularly in connection with the ghettoisation and systematic murder of the Jewish population in the eastern territories.^15^ A meticulous study has also recently been published on Hermann Eyer (1906–97), the head of the Institute for Typhus and Virus Research of the OKH,^16^ who, like Mückter, was broadly accused of involvement in human experimentation at Buchenwald.^17^ However, there is still no in-depth scholarly analysis of Mückter’s role in German typhus control and vaccine research. In most of the studies on Nazi typhus control and research cited above, he is not even mentioned by name, with the exception of the works by Schütz and Elsner. This article aims to fill this research gap: after providing a brief biographical sketch, we reconstruct Mückter’s activities during the Second World War using a variety of historical sources and situate them within the context of what Schütz has termed the ‘Nazi typhus complex’ (*nationalsozialistischer Fleckfieberkomplex*).^18^ With regard to the question of human experimentation conducted by the Institute for Typhus and Virus Research of the OKH, our findings draw primarily on Polish investigative files on Hermann Eyer and Heinrich Mückter,^19^ which have not yet been examined in German- or English-language scholarship. Moreover, drawing on a wide range of additional sources, we critically re-evaluate the overall extent of cooperation in vaccine testing and production between Eyer’s institute and the research station at Buchenwald concentration camp.

## Heinrich Mückter: a brief biographical outline

**Figure 1.**
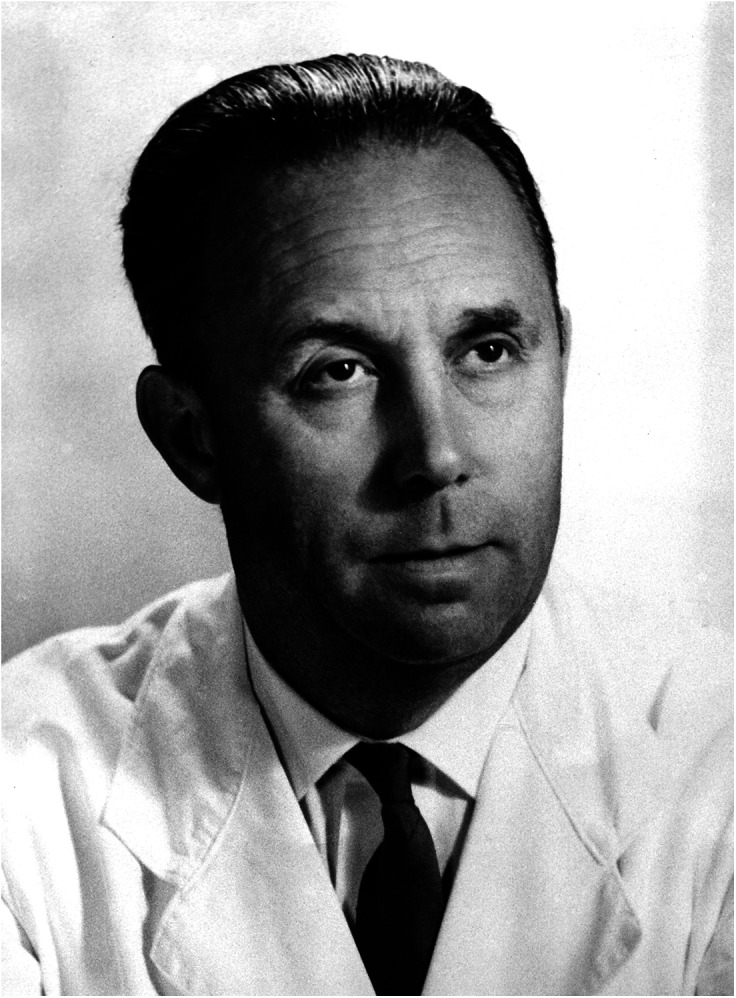
Dr Heinrich Mückter, 1968. Picture alliance | dpa.

Heinrich Mückter (Figure 1) was born on 14 June 1914 in Körrenzig, a small village in what is now the district of Düren near Aachen, the son of the road mender Heinrich Mückter (b. 1879) and his wife Margarete (b. 1891), née Jäger. He was one of eleven children. While still a student at the public grammar school in Jülich, Mückter joined the Nazi Party’s (*Nationalsozialistische Deutsche Arbeiterpartei*, NSDAP) paramilitary organisation, the *Sturmabteilung* (SA), in May 1933 – reportedly out of youthful enthusiasm. After graduating (*Abitur*) with distinction in 1934, he served in the Reich Labour Service (*Reichsarbeitsdienst*) in Goch on the Lower Rhine between May and October of the same year. From the winter semester of 1934 to the summer semester of 1939, Mückter studied medicine at the universities of Bonn and Freiburg im Breisgau. His studies were co-financed by the *Reichsstudentenwerk* (Reich Student Union). In February 1940, he received his doctorate in medicine from the University of Bonn with a dissertation in physiology. During his studies in Bonn, he was initially a member of the Catholic student association *Unitas-Salia* for two semesters, before joining the Nazi Party in 1937. After completing his medical training, Mückter began studying chemistry at the University of Bonn in January 1940. He obtained his intermediate diploma (*Vordiplom*) in 1942 and, following a wartime interruption, completed his studies with honours in late 1946.^20^

During his medical studies, Mückter had already worked at the Physiological Institute in Bonn under Ulrich Ebbecke (1883–1960). Under Ebbecke’s supervision, he also completed his doctoral thesis, ‘Der Einfluß hoher Drucke auf den Ruhestrom der Froschhaut’ [The Influence of High Pressure on the Resting Current of the Frog Skin],^21^ which was awarded *magna cum laude.* In the winter semester of 1938/9, he served as an assistant at the Anatomical Institute in Bonn under Philipp Stöhr (1891–1979),^22^ a former participant in the Kapp Putsch with strong ties to leading Nazi figures such as Science Minister Bernhard Rust (1883–1945).^23^ After receiving his war-related provisional medical licence (*Notapprobation*) in October 1939,^24^ Mückter became a devoted student of the Bonn pharmacologist Werner Schulemann (1888–1975), who is widely regarded as his academic mentor.^25^ Schulemann, like Mückter later, was both a physician and a chemist. He had long served as head of research at Bayer (IG Farben) in Elberfeld before being appointed professor of pharmacology in Bonn in February 1938. Notably, he delivered his inaugural lecture in SA uniform.^26^ Under Schulemann’s supervision and with financial support from the German Research Foundation (*Deutsche Forschungsgemeinschaft*, DFG), Mückter spent approximately one year conducting research on the chemotherapy of streptococcal infections.^27^

From December 1940 to April 1945, Heinrich Mückter served in the Wehrmacht, initially as an Officer Aspirant (*Unterarzt*, ‘Junior Physician’). He was stationed in Norway from February to April 1941 and, after a brief return to the University of Bonn, was deployed in what is now Belarus and Poland from early 1942 until early 1945.^28^ Mückter was commissioned as a Medical Officer in June 1942 (*Assistenzarzt*, ‘Assistant Physician’, Second Lieutenant) and was subsequently promoted to *Oberarzt* (‘Senior Physician’, First Lieutenant) in May 1943 and *Stabsarzt* (‘Staff Physician’, Captain) in November 1944.^29^ From spring to December 1942, he served in *Heeres-Betreuungs-Kompanie 14* in Grodno (Białystok District), which operated a delousing facility until spring 1943.^30^ In December 1942, Mückter transferred to the Institute for Typhus and Virus Research of the OKH, eventually becoming head of its Krakow branch, which remained operational until just before the city’s capture by Soviet forces on 19 January 1945. For his work in vaccine production for the German Army, Mückter was awarded the War Merit Cross, Second Class (*Kriegsverdienstkreuz II. Klasse*), in 1944. On 17 January 1945, he transferred to the institute’s evacuation site in Roth near Nuremberg, where he was captured by US forces in April and held as a prisoner of war until September. After his release, he returned to the University of Bonn and completed his chemistry studies in late 1946. In June of the same year, he was appointed head of research and production at Chemie Grünenthal in Stolberg.^31^ Although the Polish judiciary had been investigating him between 1945 and 1947 – among other charges, for conducting human experiments on Polish workers – and had issued an arrest warrant, Mückter was neither apprehended by the Western Allies nor extradited to Poland.^32^

The pharmaceutical company Grünenthal was founded in January 1946 by the Wirtz family, owners of the long-established soap and detergent manufacturer Dalli-Werke Mäurer & Wirtz. In the company’s early years, Heinrich Mückter played a central role and served both as head of research and head of production; he received at least a one-percent share of profits from product sales – amounting to over 1.9 million Deutsche Mark between 1951 and 1961.^33^ Mückter had succeeded in acquiring *Penicillium* strains from Theodor Joseph Bürgers (1881–1954), professor of hygiene in Göttingen (formerly in Königsberg), whom he had met through his work at the delousing facility in Grodno, and from Hans Knöll (1913–78), head of the Institute of Microbiology at Schott in Jena. Following an initial phase of improvised small-scale production, the first liquid penicillin preparation was clinically tested on sick infants at the St Vincenz Children’s Home in Stolberg, in collaboration with Theodor Kersting (1901–76), chief physician of the affiliated children’s hospital. The test subjects – mostly malnourished infants, likely suffering from impetigo infectiosa – had frequently died within days due to sepsis. According to Mückter’s report, the trials yielded positive results, prompting him and the company heads to initiate mass production. By 1948, Grünenthal was able to manufacture penicillin on an industrial scale, enabling the company to expand significantly.^34^

Mückter’s subsequent involvement in the development and distribution of thalidomide, as well as the related legal proceedings from January 1968 to December 1970, has been thoroughly researched^35^ and cannot be addressed here. In April 1968 – while, as a defendant in the thalidomide trial, he was at the centre of national and international press coverage – Mückter was questioned by the North Rhine-Westphalia State Criminal Police Office about his activities and observations in Grodno in 1942. The interrogation was unrelated to the thalidomide trial and took place in the context of investigations directed against the two former Gestapo officers Heinz Errelis (1912–94) and Kurt Wiese (1914–87). Between 1941 and 1943, Errelis and Wiese had been centrally involved in the planning and execution of mass shootings and deportations of Jewish civilians in Grodno. Mückter, however, had no direct connection with either of the two men and was questioned only as a witness in the proceedings; any possible involvement by Mückter in (medical) crimes was not under investigation.^36^ After retiring in the mid-1970s,^37^ Heinrich Mückter died in Aachen on 22 July 1987.^38^

## Nazi wartime typhus research, epidemic control, and health policy in the occupied eastern territories

Epidemic typhus, also known as *Typhus exanthematicus* or louse-borne typhus (German: *Fleckfieber*, literally ‘spotted fever’^39^), is caused by *Rickettsia prowazekii* and transmitted by body lice, typically when their faeces enter skin abrasions or the mucous membranes of the eyes or mouth. Symptoms typically appear after an incubation period of seven to fourteen days and include sudden onset of high fever, severe headache, and profound prostration. Body temperature remains elevated (around 40°C) for approximately two weeks. On days four to six, small pink macules usually emerge, spreading across the trunk and extremities, though typically sparing the palms, soles, and face. Splenomegaly may occur, and hypotension is common among severely ill patients. The most effective preventive measures are immunisation and louse control. Before the discovery of antibiotics, the disease was fatal in ten to twenty per cent of cases.^40^

A few weeks after the German invasion of the Soviet Union on 22 June 1941, lice infestation began to spread rapidly among German troops. By December, typhus had broken out along the entire Eastern Front. During the winter of 1941/2, more than 15,000 soldiers in the Eastern Army contracted the disease; by August 1942, the number had risen to 32,718, and over the first three and a half years of the war, more than 50,000 soldiers were affected.^41^ According to the Army Surgeon, as of 20 August 1942, there were 39,913 documented cases of typhus in the Eastern Army, Ukraine, Ostland, the General Government (*Generalgouvernement*), and Königsberg^42^ – compared to just 40 cases^43^ in the West. However, it was not only soldiers in overcrowded quarters who were at risk: epidemic outbreaks also frequently occurred among civilians and in prisoner-of-war camps. In particular, concentration camps were highly susceptible to typhus due to frequent overcrowding, abysmal hygiene, and extremely poor living conditions.^44^

Against this background, in early 1942, the *Waffen-SS* (armed wing of the Nazi Party’s *Schutzstaffel*) began testing new typhus vaccines on prisoners at Buchenwald concentration camp – in close collaboration with civilian and military authorities and the pharmaceutical industry. Vaccine production under SS authority commenced in 1943/4.^45^ However, vaccine research was not limited to the Department for Typhus and Virus Research of the Waffen-SS Hygiene Institute (*Abteilung für Fleckfieber- und Virusforschung des Hygiene-Instituts der Waffen-SS*), which oversaw the Buchenwald testing station. It also involved a broad network of partly competing institutions. In addition to the aforementioned Wehrmacht facility – the Institute for Typhus and Virus Research of the OKH in Krakow – civilian institutions such as the renowned Robert Koch Institute in Berlin, the State Institute for Experimental Therapy in Frankfurt am Main, and the Warsaw branch of the Hamburg Tropical Institute were also engaged in typhus research.^46^ Vaccine trials on concentration camp inmates were not confined to Buchenwald: Eugen Haagen (1898–1972), a researcher at the Robert Koch Institute and later professor at the Reich University of Strasbourg, also conducted several series of experiments at the Schirmeck and Natzweiler-Struthof concentration camps.^47^ As a whole, the Nazi typhus complex ‘clearly show[s] the cooperation between civil medical research institutions, military medicine within the German armed forces and SS, and the pharmaceutical industry’.^48^ Gerhard Baader characterised much of the biomedical research and experimentation conducted in the concentration camps as ‘thoroughly reliable in terms of approach and purpose’.^49^ More recent scholarship has also emphasised the scientific rigor of many of these human experiments. Volker Roelcke, for instance, notes that the sulfonamide experiments in Nazi concentration camps regarding their ‘design’ and methodology ‘corresponded to the contemporary state of scientific knowledge and to the methodological standards at the time the experiments were started’.^50^ Overall, medical-historical research in recent years has underscored that most Nazi human experiments were not examples of ‘pseudoscience’, but rather followed the logic of contemporary biomedical research.^51^ Nevertheless, these experiments – carried out in the ethically unrestrained environment of the concentration camps – were unquestionably brutal medical crimes that claimed several thousands of lives by the end of the war.^52^

Although typhus vaccinations among German soldiers on the Eastern Front eventually helped reduce mortality under relatively stable frontline conditions, the vaccines initially had little impact. Quantities were insufficient and deliveries arrived far too late. As a result, medical command focused not only on immunisation but also on improving hygiene and implementing large-scale delousing operations to contain the spread of typhus. The German occupying forces pursued both the segregation of the civilian population and delousing through physical and chemical means.^53^ As Christopher R. Browning has pointed out, the persistent antisemitic stereotype of Jews as carriers of disease – rooted in medieval imagery of the ‘plague-bearing Jew’ – resurfaced in the Nazi era, with typhus replacing plague as the perceived threat. The revival of the ghetto as a supposed ‘medical solution’ was emblematic of this logic. The establishment of the Warsaw Ghetto became a model for similar policies throughout the occupied eastern territories.^54^ The association of ‘Eastern Jews’ (*Ostjuden*) with typhus – initially derived from local living conditions – was reinforced and institutionalised through the policy of mass ghettoisation, which in turn created the very conditions that promoted disease. This process, as Browning observes, became a ‘self-fulfilling prophecy’.^55^

**Figure 2.**
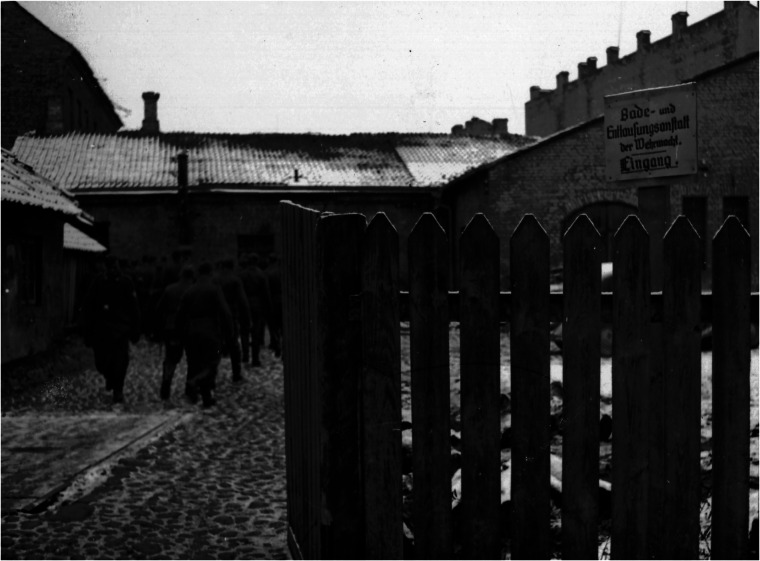
Wehrmacht delousing facility in Vilnius, Lithuania, 1940s. Private collection.

In addition to racially motivated segregation and ghettoisation measures targeting the civilian population, delousing procedures through disinfection (*Entseuchung*) and vermin control (*Entwesung*) primarily targeted German soldiers, prisoners of war, non-German auxiliary volunteers, and forced labourers. *Entseuchung* referred to the elimination of *Rickettsia*, while *Entwesung* meant the eradication of lice as disease vectors. To achieve both aims, the German authorities commonly treated clothing with hot air and steam. Chemical delousing agents were also used, including *Lauseto*, the gas *Ventox*, and *Zyklon B* (prussic acid), which would later be employed in Auschwitz for mass murder.^56^ Delousing stations (*Entlausungsanstalten*) (Figure 2) with a daily capacity of three to five thousand people were established along the East Prussian border, in the General Government, and in the occupied Soviet territories.^57^ However, in addition to segregation measures, delousing efforts also targeted local civilians, particularly those presenting with fever and headaches. These individuals were identified and registered by so-called epidemic squads (*Seuchentrupps*). Suspected typhus cases were subjected to compulsory delousing procedures: they were shaved bald, their homes disinfected with chemical agents, and their clothing deloused. During this time, suspects were forcibly hospitalised, and their close contacts were placed under a seven-day quarantine. In some regions, all non-sedentary individuals were classified as potential typhus carriers and interned in camps.^58^

As will be shown, Heinrich Mückter operated ‘between Grodno and Krakow’ in the East; that is, he played a significant role in both typhus control measures in Grodno (Białystok District, now Belarus) and in vaccine research and production in Krakow (General Government, now Poland).

## Heinrich Mückter in Grodno, Białystok District

From spring until December 1942, Heinrich Mückter worked at a delousing facility in Grodno, located in the Białystok District, together with the oto-rhino-laryngologist Josef Terrahe (b. 1901), father of Klaus Terrahe (1935–2023). The facility was primarily responsible for delousing military personnel prior to their return home, as well as foreign labourers who were being transported to the Reich for work assignments.^59^ In a brief article on the diagnosis of typhus, Mückter described his experiences with the ‘mass delousing of Russians’^60^ – carried out in what was one of the central sites of Holocaust atrocities.

The Białystok District was a site of open, genocidal violence against the Jewish and Polish civilian population, where the mass extermination of Jews – the so-called Holocaust by bullets – began earlier than in many other regions. By the time the Red Army liberated the area in July 1944, approximately 210,000 Jews and 72,000 Poles had been murdered there.^61^ The perceived risk of typhus served the occupiers as a pretext for violence, functioning as what Paul Weindling has termed an ‘incitement to kill on medical pretexts’.^62^ In early November 1942, the Jewish population across the entire Białystok District was forcibly confined to ghettos. In Grodno, two large ghettos had already been established in November 1941: Ghetto I for Jews deemed fit for labour, and Ghetto II for those classified as unfit. Between late November and early December 1942, at least five thousand people were deported from Grodno to Auschwitz, where most were murdered.^63^ Earlier that year, Reserve Police Battalion 91 had already shot elderly and sick people as actual or potential carriers of typhus.^64^

Heinrich Mückter later claimed to have been unaware of these mass shootings and extermination transports. During the 1968 interrogation, he recalled the two ghettos and admitted that Jewish forced labourers had been employed at the delousing facility. One of them was his laboratory assistant, the (former) medical student Henryk Bronstein, who lived in the ghetto and suddenly stopped coming to work one day. According to Mückter, Bronstein must ‘have been taken away at that time’. On the record, Mückter stated that he had ‘no knowledge of any deportations from the ghettos in Grodno or even acts of murder against Jews’.^65^ This statement, however, is clearly contradicted by the testimony of Mückter’s orderly, Friedrich Grau (b. 1905), and the nurse Karl-Heinz Herling (b. 1920), which indicates that both the ongoing shootings and the sealing off of the ghettos, as well as the large deportation transports and their destination – Auschwitz – were generally known to the staff of the delousing facility.^66^ It is noteworthy that the delousing facility was dissolved in the spring of 1943^67^ – shortly after Mückter’s transfer to Krakow – at a time when both ghettos had already been liquidated and the remaining Jewish population deported to the Białystok ghetto or to extermination camps.^68^ This temporal proximity suggests that the work of the delousing facility was at least *indirectly* connected to the ghetto system and its guard structures.

**Figure 3.**
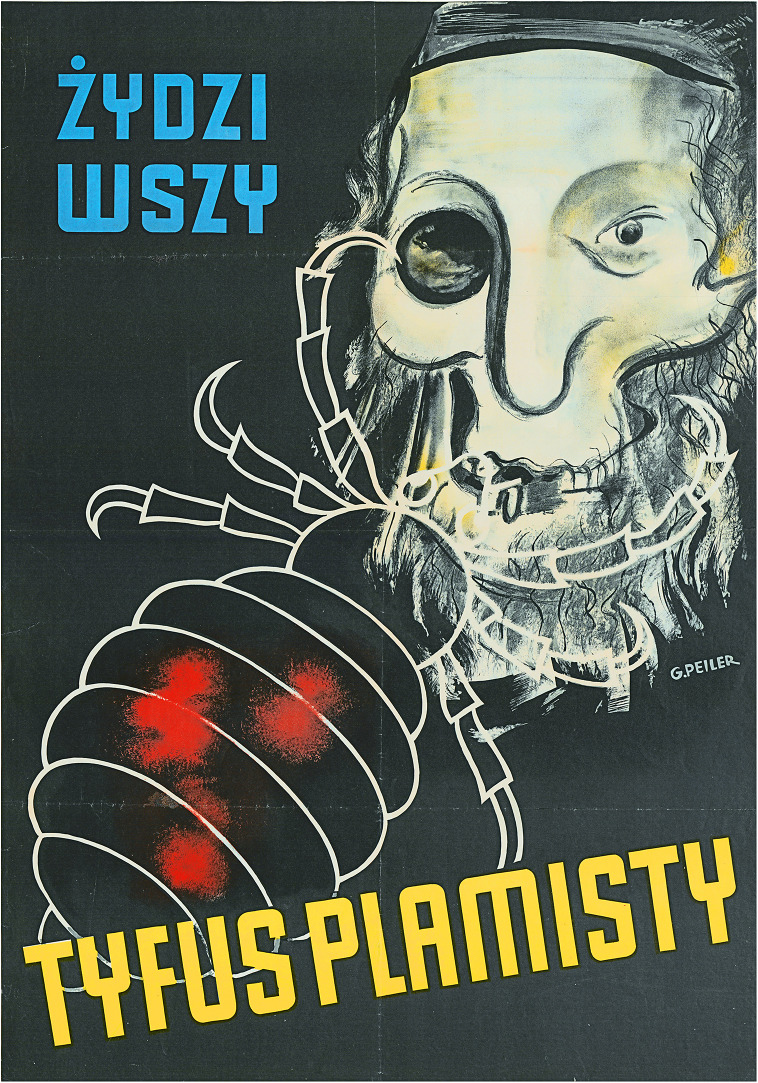
‘Jews – lice – spotted typhus’. Antisemitic propaganda poster issued by the German occupation authorities in the General Government, featuring an illustration by G. Peiler, 1941. Biblioteka Jagiellońska, 804981 V, T.3/61(4). Public domain.

Jost Walbaum (1889–1969), *Gesundheitsführer* (‘Health Leader’) in the General Government, made it unequivocally clear at a health policy conference in October 1941 that the spread of typhus from Jewish ghettos to German civilians and soldiers had to be prevented at all costs, even if this meant shooting Jews without hesitation. Among those present was Hermann Eyer, later Mückter’s superior in Krakow.^69^ Eyer himself promoted the most radical linkage between antisemitic and anti-Slavic stereotypes and the threat of typhus. In 1940, he asserted that the disease originated among ‘people of unhygiene’ and that typhus lice were bred in ‘filth-encrusted squalid dwellings [*schmutzstarrenden Elendsquartieren*] of Polish Jews’, in the ‘fur coat [*Kozuch*] of the Polish cottager’, and in the ‘caftan of the ghetto Jew’.^70^ In a review, Eyer explicitly endorsed Walbaum’s view that the ‘typhus question’ could be resolved most quickly and efficiently through ‘the complete removal of the Jewish population from Poland’.^71^ Against this background, and in light of the testimony of the delousing facility staff mentioned above, it seems highly implausible that Mückter was unaware of the extermination operations and their ideological and practical context. These measures were part of a broader policy that fused public health rhetoric with genocidal practice – an approach that, according to Walbaum, was openly discussed and advocated on site.^72^ Here, epidemic-control measures were intertwined with an exterminatory racism: in the wake of the failed ghettoisation strategy, segregation measures originally intended to isolate and eliminate the *pathogen* escalated into measures aimed at the extermination of the *human carrier* (Figure 3). Thus, medically indicated measures to combat typhus – and, in Browning’s terms, particularly their failure as a ‘self-fulfilling prophecy’ – played a decisive role in theoretically legitimising and practically initiating genocide in the period between 1939 and 1941/2. This path to genocide, however, was, as is widely recognised, by no means linear but rather shaped by processes of cumulative radicalisation among various actors and divergent interest groups, situational contingencies, and countervailing developments.^73^ At the same time, particularly in the fields of hygiene and bacteriology, such hierarchies of value, patterns of thought, and genocidal practices facilitated the growing ethical erosion of research conducted on those regarded as ‘racially worthless’ Slavic and Jewish ‘subhumans’ (*Untermenschen*).^74^

## Heinrich Mückter in Krakow, General Government

In 1983, Grünenthal’s company magazine stated that Mückter ‘approached problems of delousing so scientifically that, by December 1942, he had already found himself at the Institute for Typhus and Virus Research of the Army High Command in Krakow’.^75^ A laboratory had originally been established at the University of Krakow in November 1939 to produce a typhus vaccine exclusively for the German Wehrmacht. In early 1940, it was expanded into the Institute for Typhus Research – later renamed the Institute for Typhus and Virus Research – of the OKH (until June 1940 of the *Oberkommando der Wehrmacht*, OKW).^76^ Additional branches followed: Lviv (Lwów, Lemberg) in July 1941 and Rabka in the Tatra Mountains in early 1943. In April 1944, the Lviv branch was evacuated to Częstochowa (Tschenstochau) and Krakow. While the newly established branch in Częstochowa had fallen into the hands of the Red Army, the Rabka branch and the section of the Krakow main facility originating from Lviv had been relocated to Roth, near Nuremberg, by August 1944. Following the complete evacuation from Krakow to Roth in January 1945, the institute was handed over to American troops on 20 April 1945. The Krakow main facility was originally divided into five technically and organisationally coordinated departments: the Lice Breeding Department, Lice Infection Department, Lice Preparation Department, Vaccine Production Department, and Vaccine Control and Dispatch Department.^77^ By the end of the war, over three million vaccine doses had been manufactured from lice intestines^78^ – according to other estimates, as many as 4.5 million.^79^

Having initially served as head of the Vaccine Production Department, Heinrich Mückter ultimately took over as head of the Krakow branch of the institute – most likely in August 1944, following the relocation of his superior, Hermann Eyer, the director of the overall institution, to Roth.^80^ Born in Mannheim in 1906, Eyer studied medicine and chemistry in Heidelberg, where he earned doctorates in both fields. He joined the SA in 1933 and the Nazi Party in 1935, completed his postdoctoral qualification (*Habilitation*) in hygiene and bacteriology in Erlangen in 1936, and from 1937 was affiliated with the Military Medical Academy in Berlin, which assigned him to the Robert Koch Institute for further training. In early 1940, Eyer established vaccine production in Krakow, focusing on the so-called Weigl vaccine, developed by the Austrian-Polish biologist Rudolf Weigl (1883–1957),^81^ which was derived from the intestines of lice. In 1943, he was appointed adjunct professor (*außerplanmäßiger Professor*) at the Institute of Hygiene of the University of Berlin.^82^ Together with Mückter, Eyer conducted four experimental studies – most notably a study on the value assessment of typhus vaccines – for which protocols were kept, although they were never finalised for publication due to time constraints.^83^ The manuscripts are now considered lost.^84^

**Figure 4.**
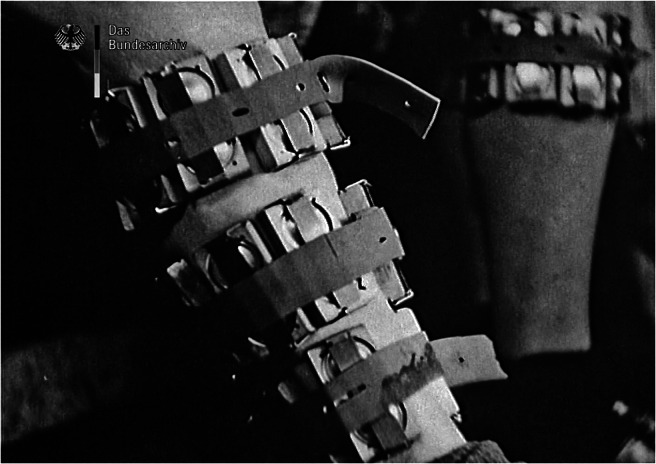
Louse-feeders at the Krakow institute with so-called louse cages attached to their lower legs, March 1941. Film still from *Kampf dem Fleckfieber!* Bundesarchiv, Film 4273, min. 21:38.

The production of the Weigl vaccine involved an extremely complex process that depended on the participation of a large number of individuals. Eyer reportedly employed up to two thousand Polish workers – what Paul Weindling called ‘a small army’.^85^ These individuals served as ‘infectors’ (*Infektoren*) and ‘louse-feeders’ (*Läusefütterer*).^86^ The complete procedure is shown in detail in the educational and propaganda film *Kampf dem Fleckfieber!* [Fighting Typhus!], produced in 1941.^87^ In Krakow, specially bred body lice were initially infected per-anally with *Rickettsia* bacteria, which then multiplied in living tissue. For several days, the lice were fed on human blood by the louse-feeders (Figure 4). Their intestines, filled with *Rickettsia*, along with their excrement – also containing pathogens – were then emulsified in a phenol-saline solution. This process inactivated the bacteria while preserving their antigenic properties, resulting in an inactivated vaccine against typhus.^88^ Louse feeding weakened the feeders physically and increased their susceptibility to infectious diseases.^89^ According to Weindling, the Germans regarded this work as ‘a task for racial inferiors’.^90^ Eyer, Mückter, and other institute personnel later testified that the louse-feeders had volunteered and received a fixed salary, food, and performance-based cash bonuses.^91^ A US military report likewise stated that there were ‘always plenty of towns people’ willing to volunteer.^92^ This notion of ‘voluntariness’ under occupation must be seen in context: Polish louse-feeders were generally shielded from arbitrary arrest and persecution, as they were classified as essential war workers – a status that placed them under a precarious form of protection.^93^

The Polish bacteriologist Zdzisław Przybyłkiewicz (1908–96) offered a markedly different account of conditions on site, including the role of Heinrich Mückter. Like Rudolf Weigl, who continued his work at ‘his institute’ – formally the Lviv branch of the OKH institute headed by Eyer’s deputy Josef Daniels (1910–83) – Przybyłkiewicz had worked closely with Hermann Eyer during the war; his testimony led to Eyer’s brief imprisonment by British authorities. He accused Eyer of looting and destroying equipment at the universities of Krakow and Lviv, and of subjecting Polish subordinates to harsh treatment and disproportionate punishments for minor infractions.^94^ He made similar allegations against Mückter, stating that he had stolen materials from the Krakow Institute of Bacteriology and transferred them to Germany. In his testimony, Przybyłkiewicz further asserted:

While working with the German Institute Mückter was very brutal to the Polish personnel, insulting them, very often striking. But he has been particularly brutal to the patients. I had seen him on many occasions forcing patients who had typhus and 40°C of temperature to walk and to wait for him in his waiting room for hours. This treatment resulted in death of some of the patients. I saw him twice kicking a patient out of his bed as the man was too weak to get up.^95^

However, as Mathias Schütz notes, Przybyłkiewicz’s credibility was already being questioned during the investigations against Eyer. The scientist appears to have enjoyed a privileged position with the German authorities due to his expertise in Weigl vaccine production, while many of his Polish colleagues were deported. It is conceivable that Przybyłkiewicz sought to obscure his own wartime collaboration in the ‘tense Polish post-war atmosphere’ and therefore deliberately incriminated former colleagues – Mückter among them – with particular severity.^96^

While Schütz offers only a cautiously affirmative answer to the question of whether human experiments were conducted in Krakow,^97^ the Polish investigation files include original documents from the institute that provide unequivocal proof of such experiments. Specifically, they contain two detailed reports by Mückter, dated 15 November and 30 December 1944, concerning experiments with trench fever pathogens conducted on Polish louse-feeders between September and December 1944.^98^ Trench fever (*Schützengrabenfieber*, *Wolhynisches Fieber*, *Fünftagefieber*, or *Quintana* in German) is a louse-borne disease caused by *Bartonella quintana* (originally known as *Rickettsia quintana*). It was first observed among soldiers during the First World War (1914–8). The disease typically manifests with intermittent fever, headache, and limb pain, most notably symmetrical pain in the tibiae. Splenomegaly is consistently observed during the acute phase; both early and late relapses may occur. Infections frequently occurred among the louse-feeders during the production of the Weigl vaccine, caused by exposure to lice infected with *Bartonella quintana.* ^99^

According to the reports, Mückter conducted two series of human experiments to test a vaccine against trench fever. An initial trial on guinea pigs was taken to indicate that laboratory animals were unsuitable for such experiments. However, Mückter reported having demonstrated evidence of immunity in a self-experiment with experimental infection, carried out in September 1944 together with three other institute employees who had previously contracted the disease, using two newly recruited louse-feeders as a control group. Encouraged by these results, he initiated a first series of experimental infections involving an unknown number of test subjects from among new louse-feeders who had no history of either typhus or trench fever. Mückter claimed that full consent had been obtained from the participants – a claim that must be questioned in light of the testimony of one of those affected.^100^ Schütz comments: ‘The long tradition of medical self-experimentation turned into an escalation of research on humans during the Nazi era’.^101^ Particularly revealing is Mückter’s rationale for using new louse-feeders as test subjects: according to him, the infections served *economic* purposes – as a kind of side experiment during ongoing typhus vaccine production. It also highlights the ethically questionable practices involved in vaccine production. He wrote:

The fact that every newly recruited blood donor [ie., louse-feeder] in the breeding program sooner or later went through their trench fever, resulting in undesirable losses of lice for us and financial loss for the individual, provided the rationale for experimentally immunising a number of blood donors, following typhus vaccination, through experimental quintana infection before the start of feeding.^102^

In a second series of trials on the prophylaxis and treatment of trench fever, fifty-five healthy female test subjects aged fifteen to forty-four – newly recruited as louse-feeders – were infected subcutaneously with an emulsion prepared from *Bartonella quintana*-rich lice intestines. At various time points – prior to infection in the prophylaxis trial – an antigen derived from *Bartonella quintana*-containing lice faeces was administered. In addition, the use of *Pyrifer* (a fever-inducing agent containing inactivated *Escherichia coli*) and a combination of antigen and *Pyrifer* were tested. All variations, however, led to the sobering conclusion that effective prophylactic immunisation using inactivated pathogens could not be achieved, and none of the three treatment methods demonstrated any significant therapeutic benefit. Mückter summarised the clinical presentation of the previously healthy test subjects as follows:

At the centre of the individually shaped course of illness are the periodically recurring fever episodes and the neuralgia-like complaints, which typically fluctuate in intensity at the same time. […] The sick individual has become a chronic excretor of Rickettsiae [ie., *Bartonella quintana*] in urine and lice. Subjectively, a certain fatigue and, in some cases, persistent neuralgia serve as reminders of the illness.^103^

Notably, a study conducted immediately after the end of the war suggests that human experiments involving artificial infection would, from the outset, have been unnecessary, as the question of immunisation against trench fever by vaccination could already have been answered in rabbits: independently of Mückter’s results, it demonstrated that, unlike in the case of typhus, vaccination offered no substantial protective efficacy.^104^ Mückter’s experiments, however, were not only methodologically questionable but ethically indefensible in several respects – even by the standards of the time. This applies, for example, to the official but largely ineffective^105^ research guidelines issued by the Reich Ministry of the Interior in 1931. These guidelines stipulated that any experiment on human beings must be rejected ‘if it can be replaced by an experiment on animals’ and may only be undertaken once comprehensive evidence has been obtained by means of laboratory and animal experiments. They further declared that scientific experiments involving individuals under the age of eighteen were inadmissible ‘if they endanger the child or adolescent even in the slightest’.^106^ Even in the criminal vaccine trials conducted at the Buchenwald concentration camp, all test subjects were at least twenty years old.^107^ The guidelines also explicitly referred to the heightened risks associated with experiments ‘involving living microorganisms, in particular living pathogens’. Ultimately, they emphasised that the conduct of an experiment ‘in the absence of consent is under all circumstances inadmissible’ and that ‘medical ethics reject any exploitation of a person’s social distress’ for the purpose of scientific experimentation.^108^ As with the lice-breeding and feeding program, the question of voluntariness must again be assessed against the backdrop of the overall coercive conditions faced by the Polish population in the General Government. Individuals volunteered only to the extent that a participation offered relative protection from arrest and violence under the occupation regime. Although the experiments were not conducted under direct physical coercion, they were nevertheless carried out through the systematic exploitation of this underlying state of ‘social distress’ – and most likely without explicit informed consent from the test subjects, who had originally been recruited solely as louse-feeders. However, for those deemed ‘racially inferior’, such standards simply had little, if any, validity within the occupiers’ racist hierarchies of value and utilitarian patterns of thought. Nonetheless, Mückter’s explicit reference to the alleged consent of the test subjects in his report suggests at least *some* awareness of the problematic nature of coerced research. If one also considers the remarkably brief section of the report dealing with the initial, unsuccessful animal trials on guinea pigs – which reads more like a perfunctory justification for the subsequent human experiment – it appears that Mückter was indeed familiar with the formal requirements for human experimentation set out in the 1931 guidelines.

As is evident from an article by Rudolf Weigl published in early 1947, Eyer’s institute conducted human experiments not only on trench fever but also on typhus vaccines: ‘A comparative experimental study on the efficacy of all three types of typhus vaccine [ie., louse, egg-yolk, and mouse-lung vaccine] was carried out in this Institute not only on laboratory animals but also on men’.^109^ Although the individual motives of the actors involved – Weigl among them – were certainly pluralistic, from a structural perspective, the trials to assess and optimise the protective efficacy of different typhus vaccines ultimately contributed indirectly to sustaining the anti-Slavic war of conquest and annihilation in the East. The same applies to the unsuccessful series of trench fever vaccine trials, as becomes evident from the rationale that Mückter explicitly mentioned in his report. The immunisation of the louse-feeders against trench fever was by no means meant to protect the health of the Polish workers; rather, it was intended solely to prevent production losses in the manufacture of the Weigl typhus vaccine. The workers were treated merely as a cheap and interchangeable human resource in the production process, whose health and adequate nourishment (‘financial loss for the individual’) were of concern only insofar as they ensured high output and greater efficiency – and thus, ultimately, the preservation of the occupiers’ purported supremacy.

## Heinrich Mückter and the typhus vaccine trials on inmates of Buchenwald concentration camp

On 18 December 1943, the renowned medical journal *The Lancet* published a review^110^ of a paper entitled ‘Über die Schutzwirkung verschiedener Fleckfieberimpfstoffe beim Menschen und den Fleckfieberverlauf nach Schutzimpfung’ [On the Protective Effect of Different Typhus Vaccines in Humans and the Course of Typhus after Vaccination],^111^ from the Hygiene Institute of the Waffen-SS. The reviewer noted that its (alleged) author, a certain Erwin Ding,^112^ gave his military rank as *SS-Sturmbannführer* (a mid-level SS officer rank equivalent to major), and that the study must have involved several hundred individuals whose exact day of infection he knew and whose incubation period was remarkably short. It concluded with the remark: ‘Thus, it seems that particularly heavy infections occurred in some hundreds of persons on known days during the investigations of a storm-troop leader. We leave our readers to make their own deductions’.^113^ As this episode illustrates, there was already suspicion abroad by 1943 that large-scale human experimentation with deliberate infection was taking place in German camps – although it was presumably prisoners of war, rather than concentration camp inmates, who were initially assumed to be the test subjects.^114^

Erwin Ding (1912–45) served as head of the typhus research station at Buchenwald concentration camp from early 1942. From 1943 onwards, the facility operated as part of the Buchenwald Department for Typhus and Virus Research of the Waffen-SS Hygiene Institute. Ding was born in Bitterfeld in 1912 as the illegitimate son of colonial physician Carl Freiherr von Schuler (1876–1934) and was adopted by the merchant Heinrich Ding (1867–1936). In 1944, he formally took his biological father’s surname – though without the aristocratic title – and henceforth called himself ‘Schuler’. He had previously served as an SS camp physician at Buchenwald in 1938 and 1939. After brief assignments with the SS Death’s Head Division and the SS Medical Academy in Graz, as well as bacteriological training at the Hygiene Institute of the University of Graz (followed in 1942/3 by further training at the Pasteur Institute in occupied Paris), he returned to Buchenwald at the end of 1941 on a ‘scientific mission’ from SS chief hygienist Joachim Mrugowsky (1905–48).^115^

According to research by Paul Weindling, nine typhus vaccine trials were conducted under the direction of Erwin Ding-Schuler^116^ between early 1942 and late 1944, involving a total of 480 test subjects who were artificially infected with typhus – 92 of whom died as a result. These figures do not include preliminary trials, therapeutic studies, and the infamous ‘typhus passages’ (*Fleckfieber-Passagen*) beginning in April 1943, in which individuals were deliberately infected solely to propagate the pathogen (instead of using animal passages in human-fed lice, as practised in Krakow). In total, Weindling estimates that approximately 1,000 prisoners were subjected to typhus-related experimentation, including those used for passage purposes and therapeutic studies. Of the 590 victims identified by name, 116 died as a direct consequence of the experiments.^117^ At the core of the main trials was the objective ‘to evaluate various vaccines for their ability to induce immunity with acceptable side effects’.^118^ The symptoms reported among the abused prisoners were diverse. According to Weindling and Farrell, primary symptoms included ‘fever, rash, severe headache, limb and back pain, weight loss, conjunctivitis, nosebleeds, facial swelling, paralysis, pain and swelling of the spleen, liver, and kidneys, cognitive impairment, cardiac and circulatory dysfunction, and fluctuations in eosinophil and lymphocyte counts’.^119^

**Figure 5.**
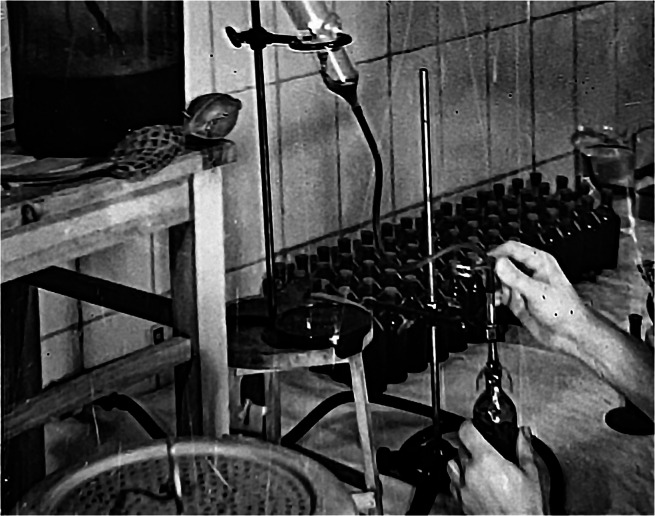
Original photograph documenting the bottling of the ‘Weimar’ vaccine in the laboratory of the Department for Typhus and Virus Research of the Hygiene Institute of the Waffen-SS at Buchenwald concentration camp, 1944. Rijksarchief in België, Dienst Archief Oorlogsslachtoffers, R 696/Tr 255612/1547. Figure 5. long description.

Let us return to Heinrich Mückter. Was he involved in the aforementioned experiments, as has frequently been claimed – albeit without supporting details or evidence? To anticipate the answer: There is no evidence or indication that Mückter ever personally visited the Buchenwald research station or had direct contact with those responsible for the experiments. His connection was indirect: among the typhus vaccines tested in Buchenwald was the Weigl vaccine produced by Eyer’s OKH institute in Krakow. According to Eugen Kogon, the OKH vaccine was considered the most effective – although extremely labour-intensive to produce – and was ‘used for control purposes’ in the comparative trials.^120^ It can be reconstructed that the Krakow vaccine was used in three of the nine trial series: the first, beginning in January 1942 (Trial Series I; 5 fatalities, none in the group receiving the Weigl vaccine); a failed comparative study from May 1943 (Trial Series VII; 53 fatalities, including 9 in the Weigl group); and the final series from July 1944 (Trial Series IX; 24 fatalities, including 5 in the Weigl group). However, the already established OKH Weigl vaccine was not the focus of the experimental inquiry; as Kogon clearly noted, it served instead as a benchmark for the evaluation of newer and more economical preparations. In addition to the SS’s own ‘Weimar’ vaccine (Figure 5) – produced using mouse and rabbit lungs following the method developed by the French researchers Paul Durand (1886–1960) and Paul Giroud (1898–1989), and successfully tested against the Weigl product in July 1944 – the trials included, among others, novel preparations developed by the Robert Koch Institute and by the Behringwerke in Marburg (IG Farben), which were produced using incubated chicken eggs, based on modified versions of the method introduced by the American researcher Herald R. Cox (1907–86).^121^

Although he did not initiate the testing of the OKH Weigl vaccine at Buchenwald himself, and the provision of the ampoules took place directly via the Hygiene Institute of the Waffen-SS and the Army Medical Inspectorate (*Heeres-Sanitätsinspektion*), it is documented that Hermann Eyer received a written report on the results of the first trial series (including the fatalities) from SS chief hygienist Joachim Mrugowsky. Thus, it is almost certain that Heinrich Mückter was also informed about the test results via Eyer. However, to conceal the deliberate infection of concentration camp inmates, Mrugowsky claimed in his report that the trials had been carried out during a typhus epidemic.^122^ At the same time, records of Erwin Ding-Schuler indicate that from at least October 1943 onwards there was no contact or collaboration between Ding-Schuler’s SS department at Buchenwald and Eyer’s OKH institute in Krakow, except for a single instance in the spring of 1944.^123^ A diary entry dated 3 May 1944 records that a courier (the department’s SS medical orderly August Feld) had ‘returned from Krakow with rickettsia-infected mouse lungs from the Typhus Institute of the OKH’.^124^ The rickettsial strains obtained from the mouse lungs were used in Buchenwald for the production of the forthcoming SS vaccine.^125^ By contrast, a telegram from Ding-Schuler to Mrugowsky further underscores that the OKH Weigl vaccine used comparatively in human trials was not obtained directly from Eyer in Krakow, but through the Waffen-SS Hygiene Institute, and that Ding-Schuler had to request and even press for its delivery.^126^ Overall, Eyer and Mückter appear to have had no direct influence on the experiments and likely kept their distance from the affairs in Buchenwald – not least due to the rivalry between the Wehrmacht and the SS in typhus vaccine research and production, personified in the antagonism between Eyer and Ding-Schuler.^127^ Notably, by the end of the war, Eyer’s OKH institute was no longer solely producing the Weigl vaccine derived from lice intestines. In 1943, under the direction of Ralf Bickhardt (b. 1912), it began manufacturing vaccines from both mouse lungs and egg yolk sacs – primarily at the branch facility in Rabka, which had been specially established for this purpose earlier that year.^128^ However, these novel OKH vaccines were evidently not included in the Buchenwald trials. Nonetheless, Eyer’s departure from his earlier categorical rejection of alternatives to the Weigl vaccine may also have been prompted by the positive results of the first two trial series conducted on 204 test subjects from January to April and August to November 1942^129^ at Buchenwald. While several trial series failed from late 1942 onwards, these initial trials confirmed the results of earlier experimental tests on guinea pigs^130^ and, for the first time, systematically demonstrated a comparable efficacy and better tolerability of the now-optimised egg-yolk and lung preparations in human subjects.^131^ As evidenced by Weigl’s aforementioned account and earlier trials aimed at reducing the dosage of the Weigl vaccine, these findings were subsequently substantiated at the OKH institute through further comparative trials with the new preparations on Polish louse-feeders.^132^

Based on Ernst Klee’s publications, research has discussed a visit by Hermann Eyer to the Buchenwald concentration camp in February 1943^133^ – a visit that Klee, in his later works, erroneously associated with the testing of the Krakow Weigl vaccine.^134^ In fact, no trials involving the OKH *typhus* vaccine were conducted at Buchenwald during this period.^135^ As Klee had correctly noted earlier, Eyer’s visit to the research station was instead related to a series of *yellow fever* vaccine trials.^136^ Notably, these trials were conducted on behalf of and in close cooperation with the Army High Command. They involved an assessment of the tolerability of different yellow fever vaccines intended for the German Africa Corps. Since the test subjects were not deliberately infected with the virus, these trials were, in terms of their criminal nature, at least not directly comparable to the often lethal experiments carried out to evaluate the efficacy and tolerability of new typhus vaccines. The first three of eleven trials commenced on 11 and 13 January 1943. A total of 85 inmates were vaccinated: Trial 0 comprised 50 individuals who received a previously tested vaccine batch from the Robert Koch Institute to assess its effect on working capacity, while Trials I and II involved 35 individuals who were administered three untested vaccine batches from the Behringwerke and five from the Robert Koch Institute (each batch tested on three to five subjects). After eight further trials, the series was discontinued on 17 May 1943 due to a war-related halt in vaccine production following the Axis defeat in the North African campaign. By then, approximately 500 subjects had been used in the test series.^137^

Together with Bernhard Schmidt (1906–2003), a hygienist from the Army Medical Inspectorate, Eyer visited the research station on 8 February 1943.^138^ In the post-war period, both claimed that the visit had merely served to demonstrate a new vaccine ampoule and the technique for opening it.^139^ It seems possible that such an ampoule-opening technique was indeed demonstrated during their visit to Buchenwald.^140^ However, Eyer and Schmidt concealed the broader context in which such a demonstration may have taken place: the commencement of Yellow Fever Vaccine Trial V, in which an OKH vaccine from Krakow was tested.^141^ On 25 February 1943, the first inmate – who had been vaccinated with the Krakow preparation on 8 February – died.^142^ Although the actual purpose of the visit had indeed been different, during his stay in Buchenwald Eyer demonstrably also inspected typhus test subjects and was shown medical records and fever charts.^143^ These subjects had been artificially infected as part of the concurrent (later failed) Typhus Vaccine Trial Series V, in which a modified Cox vaccine provided by the Behringwerke was tested.^144^ From February 1943 at the latest, Eyer must therefore have known from his own observations that the infections in the previous SS trials had not, as Mrugowsky had claimed in his 1942 report, occurred during a typhus epidemic, but that they were the result of coerced research on concentration camp inmates.

## Conclusion

As a physician at the delousing facility in Grodno, in the Białystok District, Heinrich Mückter was part of a typhus control programme heavily shaped by racist and antisemitic stereotypes – a programme whose exterminatory consequences he witnessed first-hand in what was a ‘hotspot’ of the Holocaust. As a scientist in Krakow, he was directly involved in at least one ethically problematic method of vaccine production and in highly unethical and methodologically questionable human experimentation on the Polish population. However, there is no evidence linking him to direct involvement in the criminal typhus experiments conducted at the Buchenwald concentration camp. Nonetheless, during his typhus-related activities in the Second World War, Mückter operated within different ‘Nazi spaces of violence’: the General Government and the territories targeted by the *Generalplan Ost* (‘Master Plan for the East’) – the two principal spaces for testing and implementing the Holocaust.^145^ Just as typhus research conducted within the Reich territory (first space) was linked to the more radically unrestrained vaccine research and production practices in the General Government (second space) – with overlap occurring in concentration camps as inherently unbounded spaces – the typhus program as a whole was embedded within the genocidal health and population policies pursued in the General Government and the area targeted by the *Generalplan Ost* (third space). As Paul Weindling writes: ‘Preventive medicine, research, and extermination were intertwined’.^146^ The unifying ideological framework behind health *and* extermination policy across all *three* spaces of violence was a proclaimed geo-medical approach to epidemic control that linked disease, territory, and culture.^147^ While Mückter may not have committed acts of *open* personal violence or ever set foot in a concentration camp or its affiliated research facilities, he was – like his superior Hermann Eyer – part of a far-reaching network of civilian and military scientists, health officials, and representatives of the pharmaceutical industry who accelerated the erosion of ethical boundaries in biomedical research and contributed to an exterminatory health and population policy that formed part of the German war of annihilation in the East and ultimately culminated in the Holocaust.

## Footnotes

1

Beate Kirk , Der Contergan-Fall: Eine unvermeidbare Arzneimittelkatastrophe? Zur Geschichte des Arzneistoffs Thalidomid (Stuttgart: Wissenschaftliche Verlagsgesellschaft, 1999); Trent Stephens and Rock Brynner , Dark Remedy: The Impact of Thalidomide and its Revival as a Vital Medicine (Cambridge, MA: Perseus, 2001); Arthur Daemmrich , ‘A Tale of Two Experts: Thalidomide and Political Engagement in the United States and West Germany’, Social History of Medicine, 15, 1 (2002), 137–5812625358 10.1093/shm/15.1.137; Niklas Lenhard-Schramm , Das Land Nordrhein-Westfalen und der Contergan-Skandal: Gesundheitsaufsicht und Strafjustiz in den ‘langen sechziger Jahren’ (Göttingen: Vandenhoeck & Ruprecht, 2016); Thomas Großbölting and Niklas Lenhard-Schramm (eds), Contergan: Hintergründe und Folgen eines Arzneimittel-Skandals (Göttingen: Vandenhoeck & Ruprecht, 2017).

2 Armin D. Steuer, ‘Braune Vorgeschichte: Der Contergan-Erfinder’, *Der Spiegel*, 19 November 2007, https://www.spiegel.de/geschichte/braune-vorgeschichte-a-948837.html (retrieved 1 July 2025). See also Armin D. Steuer, ‘Contergan-Erfinder kooperierte mit KZ’, *taz am Wochenende*, 6 February 1999, https://taz.de/!1303181/ (retrieved 1 July 2025). Mückter was already linked to human experimentation at Buchenwald in contemporary international press coverage of the thalidomide trial. See Anonymous, ‘The Thalidomide File: The Story of Horror Began 14 Years Ago’, *The Times-Union*, 27 May 1968.

3 Lenhard-Schramm, *op. cit.* (note 1), 47. Two laboratory reports provide conclusive evidence that the active ingredient thalidomide was developed in the laboratories of the company Grünenthal in the spring of 1954. See Wilhelm Kunz, ‘Bericht für den Monat April 1954’, 7 May 1954, Herbert Keller, ‘Bericht für den Monat Mai 1954’, 8 June 1954, Unternehmensarchiv der Grünenthal Pharma GmbH & Co. KG (Grünenthal Corporate Archive), GRT.0011.10863.0059, GRT.0011.10844.0099, https://www.contergan-skandal.de/wirkstoff-thalidomid (retrieved 3 February 2026).

4

Stephan Nuding , Profit vor Menschenrecht: Die Geschichte des Contergan-Verbrechens vom Dritten Reich bis heute (Heerlen: Dr. Rath Health Foundation, 2011), 18, 36–9.

5 Otto Ambros (1901–90) was an expert in chemical warfare at IG Farben.

6 Rosaleen Moriarty-Simmonds, ‘Thalidomide: Detailed History and Timeline’, https://www.rms-consultancy.co.uk/personnel/thalidomide-detailed-history-and-timeline/ (retrieved 1 July 2025). See also James Linder Jones, ‘Thalidomide: The Real Story & the First Seal Baby’, 11 March 2011, https://www.healthworldnet.com/articles/the-best-of-the-best/the-first-seal-baby-the-real-story-of-thalidomide.html (retrieved 1 July 2025); Vera Sharav, ‘From the Holocaust to Thalidomide: A Nazi Legacy’, 10 January 2014, https://ahrp.org/from-the-holocaust-to-thalidomide-a-nazi-legacy/ (retrieved 1 July 2025). An equally unfounded variation of this theory appears in the report on a scientific symposium organised by the German Contergan Network in 2024. See Contergannetzwerk Deutschland e.V., ‘Bericht über das Contergan-Symposium am 16.03.2024 im Großen Kursaal in Stuttgart-Bad Cannstatt’, https://www.contergannetzwerk.de/index.php/contergan-symposium.html (retrieved 1 July 2025): ‘The key conclusion of the historical section [of the symposium] was that it remains questionable whether the active ingredient in the drug Contergan was in fact developed by the company Grünenthal, as is commonly claimed, or whether it may instead have originated from the typhus vaccine programme directed by Mückter’.

7 Lenhard-Schramm, *op. cit.* (note 1), 135–6; Ann-Kathrin Hinz and Karen Nolte, ‘Historischer Rückblick auf eine beispiellose Arzneimittelkatastrophe’, in Institut für Gerontologie der Universität Heidelberg (ed.), *Contergan: Expertise zur historischen Aufarbeitung der Arbeit der Conterganstiftung*, 31 [*sic!*] April 2024, https://contergan-infoportal.de/leistungen/projekte/historische-aufarbeitung-der-arbeit-der-conterganstiftung/ (retrieved 1 July 2025), 7–29, 11–12. With reference to Auschwitz and without source citation, Julien Reitzenstein , Himmlers Forscher: Wehrwissenschaft und Medizinverbrechen im ‘Ahnenerbe’ der SS, 2nd edn (Paderborn: Schöningh, 2020), 342 : ‘The Wehrmacht therefore also conducted research into methods of combating typhus, for example by establishing the Institute for Typhus and Virus Research in Krakow under its deputy director, later head of research at the pharmaceutical company Grünenthal, Staff Physician Dr Heinrich Mückter. Here, the so-called “Weigl vaccine” was tested on prisoners from the nearby concentration camp of Auschwitz’.

8

Anne H. Crumbach , Sprechen über Contergan: Zum diskursiven Umgang von Medizin, Presse und Politik mit Contergan in den 1960er Jahren (Bielefeld: Transcript, 2018), 54 .

9

Gine Elsner , Impfen für das Dritte Reich: Über Vakzine, Versuche, Verbrechen (Hamburg: VSA, 2023), 27 . See also Sascha Topp , Geschichte als Argument in der Nachkriegsmedizin: Formen der Vergegenwärtigung der nationalsozialistischen Euthanasie zwischen Politisierung und Historiographie (Göttingen: V & R Unipress, 2013), 134 .

10

Ernst Klee , Das Personenlexikon zum Dritten Reich: Wer war was vor und nach 1945, 3rd edn (Frankfurt am Main: Fischer, 2011), 418 . See also Trent Stephens and Rock Brynner, *op. cit.* (note 1), 6–7: ‘Specifically, he was Medical Officer (*Stabsarzt*) to the Superior Command of the German Occupation Forces occupying Krakau, Poland, with the additional, ominous title of “Director for the Institute of Spotted Fever and Virus Research”. […] one can only presume that Mückter’s work there involved the science of killing rather than healing’.

11

Eugen Kogon , Der SS-Staat: Das System der deutschen Konzentrationslager (Düsseldorf: Schwann, 1946), 136 . English version published as *The Theory and Practice of Hell.* The book is based on a report compiled in April and May 1945 by Kogon and several Buchenwald inmates on behalf of the US Army. The original report has since also been published: David A. Hackett (ed.), *The Buchenwald Report* (Boulder: Westview Press, 1995).

12

Paul Weindling and Ryan A. Farrell , ‘Joachim Mrugowsky, Erwin Ding-Schuler, and Typhus Vaccine Research at “KL Buchenwald, Post Weimar”’, in Paul Weindling (ed.), Fleckfieberforschung im Nationalsozialismus: Joachim Mrugowskys Fleckfieber-Abhandlung und seine Tätigkeit als Hygieniker der Waffen-SS (Stuttgart: Wissenschaftliche Verlagsgesellschaft, 2021), 105–213, 105.

13
*Ibid.*, 106; Karl-Heinz Leven , ‘Fleckfieber beim deutschen Heer während des Krieges gegen die Sowjetunion (1941–1945)’, in Ekkehart Guth (ed.), Sanitätswesen im Zweiten Weltkrieg (Herford/Bonn: Mittler & Sohn, 1990), 127–65, 140 .

14

Christopher R. Browning , ‘Genocide and Public Health: German Doctors and Polish Jews, 1939–41’, Holocaust and Genocide Studies, 3, 1 (1988), 21–36, 2211645826 10.1093/hgs/3.1.21.

15
*Ibid.*; Ulrich Schneider and Harry Stein , IG-Farben AG, Abt. Behringwerke Marburg – KZ Buchenwald – Menschenversuche: Ein dokumentarischer Bericht (Kassel: Brüder-Grimm-Verlag, 1986); Leven, *op. cit.* (note 13); Paul Weindling , ‘Die weltanschaulichen Hintergründe der Fleckfieberbekämpfung im Zweiten Weltkrieg’, in Christoph Meinel and Peter Voswinckel (eds), Medizin, Naturwissenschaft, Technik und Nationalsozialismus: Kontinuitäten und Diskontinuitäten (Stuttgart: GNT-Verlag, 1994), 129–35; Paul Weindling , ‘Die deutsche Wahrnehmung des Fleckfiebers als Bedrohung aus dem Osten im Ersten und Zweiten Weltkrieg’, in Michael Hubenstorf et al. (eds), Medizingeschichte und Gesellschaftskritik: Festschrift für Gerhard Baader (Husum: Matthiesen, 1997), 324–3911619553; Maria A. Balińska , ‘Choroba jako ideologia: tyfus plamisty w okupowanej Polsce (1939–1944)’, Zeszyty Historyczne, 126 (1998), 212–21; Paul Weindling , Epidemics and Genocide in Eastern Europe, 1890–1945 (Oxford: Oxford University Press, 2000); Ute Caumanns and Michael G. Esch , ‘Fleckfieber und Fleckfieberbekämpfung im Warschauer Ghetto und die Tätigkeit der deutschen Gesundheitsverwaltung 1941/42’, in Wolfgang Woelk and Jörg Vögele (eds), Geschichte der Gesundheitspolitik in Deutschland: Von der Weimarer Republik bis in die Frühgeschichte der ‘doppelten Staatsgründung’ (Berlin: Duncker & Humblot, 2002), 225–62; Winfried Süß , Der ‘Volkskörper’ im Krieg. Gesundheitspolitik, Gesundheitsverhältnisse und Krankenmord im nationalsozialistischen Deutschland 1933–1945 (München: Oldenbourg, 2003); Thomas Werther, ‘Fleckfieberforschung im Deutschen Reich 1914–1945: Untersuchungen zur Beziehung zwischen Wissenschaft, Industrie und Politik unter besonderer Berücksichtigung der IG Farben’ (PhD thesis: University of Marburg, 2004); Naomi Baumslag , Murderous Medicine: Nazi Doctors, Human Experimentation, and Typhus (Westport, CT: Praeger, 2005); Klaus-Peter Friedrich , ‘Rassistische Seuchenprävention als Voraussetzung nationalsozialistischer Vernichtungspolitik: Vom Warschauer “Seuchensperrgebiet” zu den “Getto”-Mauern (1939/40)’, Zeitschrift für Geschichtswissenschaft, 53, 7 (2005), 609–36; Alexander Neumann , ‘Arzttum ist immer Kämpfertum’: Die Heeressanitätsinspektion und das Amt ‘Chef des Wehrmachtssanitätswesens’ im Zweiten Weltkrieg (1939–1945) (Düsseldorf: Droste, 2005); Alexander Neumann , ‘“Ausschaltung der Ansteckungsquellen”: Die Seuchenpolitik der deutschen Wehrmacht im Krieg gegen die Sowjetunion 1941–1944’, in Udo Schagen and Sabine Schleiermacher (eds), ‘Gesundheitsschutz für alle’ und die Ausgrenzung von Minderheiten: Historische Beiträge zur Aushöhlung eines gesundheitspolitischen Anspruchs (Berlin: Charité – Universitätsmedizin, 2006), 45–51 ; Annette Hinz-Wessels , Das Robert Koch-Institut im Nationalsozialismus, 2nd edn (Berlin: Kadmos, 2012); Arthur Allen , The Fantastic Laboratory of Dr. Weigl: How Two Brave Scientists Battled Typhus and Sabotaged the Nazis (New York, NY/London: W.W. Norton & Company, 2014); Paul Weindling , Victims and Survivors of Nazi Human Experiments: Science and Suffering in the Holocaust (London/New York, NY: Bloomsbury, 2015); Friedrich Cain , Wissen im Untergrund: Praxis und Politik klandestiner Forschung im besetzten Polen (1939–1945) (Tübingen: Mohr Siebeck, 2021); Paul Weindling (ed.), *Fleckfieberforschung im Nationalsozialismus: Joachim Mrugowskys Fleckfieber-Abhandlung und seine Tätigkeit als Hygieniker der Waffen-SS* (Stuttgart: Wissenschaftliche Verlagsgesellschaft, 2021); Elsner, *op. cit.* (note 9); Paul Weindling, ‘The Virologist Eugen Haagen (1898–1972) as a Late Nazi Election to the Leopolina of a Virologist and Professor of Hygiene at the Reichsuniversität Straßburg’, in Volker Roelcke (ed.), *Politik in der Wissenschaft: Zur Evaluierung von ‘NS-Belastung’ in wissenschaftlichen Kontexten* (Stuttgart: Wissenschaftliche Verlagsgesellschaft, 2024), 193–211.

16

Mathias Schütz , ‘Vier Ermittlungen und ein Verdienstkreuz: Der Hygieniker Hermann Eyer, der nationalsozialistische Fleckfieberkomplex und die Grenzen der Aufarbeitung’, Medizin, Gesellschaft und Geschichte, 38 (2020), 145–79.

17

Ernst Klee , Auschwitz, die NS-Medizin und ihre Opfer, 3rd edn (Frankfurt am Main: Fischer, 1997), 343–5; Ernst Klee , Deutsche Medizin im Dritten Reich: Karrieren vor und nach 1945, 2nd edn (Frankfurt am Main: Fischer, 2001), 320–1; Klee, *op. cit.* (note 10), 142.

18 Schütz, *op. cit.* (note 16), 145 *et passim.*

19 Instytutu Pamięci Narodowej w Warszawie (Institute of National Remembrance, Warsaw, hereafter abbreviated IPN), GK 164/1736 t. 1, 3.

20 Deutsche Forschungsgemeinschaft (DFG), questionnaire for Heinrich Mückter, 24 October 1939, Bundesarchiv (German Federal Archives, hereafter abbreviated BArch), R 73/13252; Military Government of Germany, questionnaire for Heinrich Mückter, 2 April 1947, Acknowledgement regarding item 45, 4 April 1947, Landesarchiv Nordrhein-Westfalen, Abteilung Rheinland (State Archives of North Rhine-Westphalia, Rheinland Department, hereafter abbreviated LAV NRW R), NW 1080 Nr. 4891; Curriculum vitae of Heinrich Mückter, undated (after 1961), LAV NRW R, Gerichte Rep. 139 Nr. 188a, fo. 17; Archive of the University of Bonn, communication on Heinrich Mückter, 27 October 2025.

21 Heinrich Mückter, ‘Der Einfluß hoher Drucke auf den Ruhestrom der Froschhaut’ (MD thesis: University of Bonn, 1939).

22 List of scientific activities of Heinrich Mückter during his studies, undated (October 1939), BArch, R 73/13252.

23

Ralf Forsbach , Die Medizinische Fakultät der Universität Bonn im ‘Dritten Reich’ (München: Oldenbourg, 2006), 79 .

24 Pharmakologisches Institut der Universität Bonn, Werner Schulemann, to DFG, 17 November 1939, BArch, R 73/13252.

25

Wilhelm Girstenbrey , ‘Pioniertaten im Stolberger Kupferhof: Der Aufstieg der Grünenthal GmbH mit dem “Wundermittel” Penicillin’, in Karl Schleicher (ed.), Stolberg nach dem Kriege 1945–1948 (Stolberg: Burg Verlag & Druck, 1988), 155–64, 157–8. Reprint of Wilhelm Girstenbrey , ‘Der Aufstieg der Grünenthal GmbH mit dem “Wundermittel” Penicillin: Pioniertaten im Stolberger Kupferhof’, Die Waage, 22, 4 (1983), 126–32.

26 Forsbach, *op. cit.* (note 23), 100–12.

27 DFG, Rudolf Mentzel, to Heinrich Mückter, 5 January 1940, DFG, Ferdinand Sauerbruch, to Heinrich Mückter, 28 March 1940, Pharmakologisches Institut der Universität Bonn, Werner Schulemann, to DFG, 6 December 1940, BArch, R 73/13252.

28 Military Government of Germany, questionnaire for Heinrich Mückter, 2 April 1947, LAV NRW R, NW 1080 Nr. 4891; Curriculum vitae of Heinrich Mückter, undated (after 1961), LAV NRW R, Gerichte Rep. 139 Nr. 188a, fo. 17.

29 Oberkommando der Wehrmacht, personnel record card for Heinrich Mückter, US National Archives and Records Administration, NAID 401141354.

30

Serge Klarsfeld (ed.), Documents concerning the Destruction of the Jews of Grodno 1941–1944, Vol. 2 (Paris/New York, NY: Beate Klarsfeld Foundation, 1985), 127–30, 150–2, 167, 188–9, 252–3; Girstenbrey, *op. cit.* (note 25), 158.

31 Institut für Fleckfieber- und Virusforschung des OKH, appointments of officers and officials in officer rank, 1 February 1945, BArch, RH 15/197, fo. 122; Joseph E. Smadel, Hans G. Schlumberger and Eugene P. Soles, *Institut für Fleckfieber- und Virusforschung des O.K.H.* (London: Combined Intelligence Objectives Sub-Committee, 1945), 3–5; Military Government of Germany, questionnaire for Heinrich Mückter, 2 April 1947, LAV NRW R, NW 1080 Nr. 4891; Heinrich Mückter to the Dean of the Medical Faculty of the University of Bonn, Erich von Redwitz, 19 November 1946, IPN, GK 164/1736 t. 1, ff. 217–21; Original English translation of the testimony by Zdzisław Przybyłkiewicz on the action sheet concerning Heinrich Mückter, 25 July 1947, IPN, GK 164/1736 t. 3, fo. 13; Curriculum vitae of Heinrich Mückter, undated (after 1961), LAV NRW R, Gerichte Rep. 139 Nr. 188a, fo. 17; Girstenbrey, *op. cit.* (note 25), 157–8; Schütz, *op. cit.* (note 16), 151. Mückter obtained the position at Grünenthal on the recommendation of Werner Schulemann, a friend of the company’s owner, Hermann Wirtz (1896–1973). A job offer from Schering in Berlin was reportedly declined by Mückter in favour of Grünenthal. See Klara van Eyll, ‘Der Hof Grünenthal und die Familie Wirtz: Vom Kupferhof zur Pharmaforschung’, *Die Waage*, 35, 2 (1996), 48–57, 54.

32 Wanted report concerning Heinrich Mückter, undated (1945/6), Action sheet concerning Heinrich Mückter, 25 July 1947, IPN, GK 164/1736 t. 3, ff. 12–15. See also Schütz, *op. cit.* (note 16), 147–54.

33 Curriculum vitae of Heinrich Mückter, undated (after 1961), LAV NRW R, Gerichte Rep. 139 Nr. 188a, fo. 17; Kirk, *op. cit.* (note 1), 51; Lenhard-Schramm, *op. cit.* (note 1), 135–6.

34 Girstenbrey, *op. cit.* (note 25), *passim*; van Eyll, *op. cit.* (note 31), 57; Holger Kraneis , ‘Aus der Nothilfe der Nachkriegszeit erwächst ein modernes Pharmaunternehmen: Pilze, Penicillin und Pioniergeist’, Die Waage, 35, 2 (1996), 58–65 ; Lenhard-Schramm, *op. cit.* (note 1), 135–6. See also Ingrid Pieroth , Penicillinherstellung: Von den Anfängen bis zur Großproduktion (Stuttgart: Wissenschaftliche Verlagsgesellschaft, 1992), 88–9, 123–4; Wolfgang Forth and Dietmar Gericke , ‘Von Menschen und Pilzen: Zur Entwicklung der Penicillinherstellung in Deutschland’, Deutsches Ärzteblatt, 92, 16 (1995), 1163–76, 1168; Wolfgang Forth , Dietmar Gericke and Ernst-Günther Schenck , Von Menschen und Pilzen: Zur Geschichte der Penicillin-Produktion im ehemaligen Deutschen Reich und in der Besatzung nach 1945 (München: Zuckschwerdt, 1997), 56–8, 64–8; English version: Wolfgang Forth , Dietmar Gericke and Reinhard Klimmek , Men & Fungi: Penicillin Research and Production in World War II Germany (München: Zuckschwerdt, 2000), 48–50, 56–9. Regarding the actors and circumstances of the production and testing of penicillin in Jena in 1945, see Florian Steger and Jan Jeskow , Ludwig Heilmeyer: Eine politische Biographie (Stuttgart: Steiner, 2021), 101–11. For pharmaceutical testing at St Vincenz Children’s Home, see now Silke Fehlemann, Sylvia Wagner and Heiner Fangerau, ‘Formen der missbräuchlichen Medikamentenvergaben am Beispiel von Aktenauswertungen verschiedener Einrichtungen’, in Heiner Fangerau *et al.* (eds), *Missbräuchlicher Einsatz von Medikamenten an Kindern und Jugendlichen in Nordrhein-Westfalen seit der Gründung des Landes bis in die 1980er Jahre* (Weinheim: Beltz Juventa, 2026), 137–230, 217–8.

35 Kirk, *op. cit.* (note 1); Lenhard-Schramm, *op. cit.* (note 1); Großbölting and Lenhard-Schramm, *op. cit.* (note 1).

36 Klarsfeld, *op. cit.* (note 30), 79–123, 188–9, 270–364; Serge Klarsfeld (ed.), *Documents concerning the Destruction of the Jews of Grodno 1941–1944*, Vol. 5 (Paris/New York, NY: Beate Klarsfeld Foundation, 1985), *passim.*

37 In 1974, Leopold Flohé (b. 1938) was appointed by Mückter as Head of the Pharmacotherapeutics Section at Grünenthal and succeeded him in 1976 as Director of Research and Development. In his memoirs, Flohé states that Mückter died of liver cancer only a few months after his own entry into the company. See Leopold Flohé, ‘Rückblenden’, May 2018, https://www.researchgate.net/publication/325083265 (retrieved 3 February 2026), 46–8, 104. In fact, however, Mückter lived for more than ten further years and appears to have retired for health reasons in 1974/5.

38 DFG, ‘Mückter, Heinrich’, *Gepris historisch 1920–1945*, https://www.gepris-historisch.dfg.de/person/5108352? (retrieved 1 July 2025). The gravestone at Aachen’s Waldfriedhof also gives this date of death.

39 In German usage, the term *Typhus* refers exclusively to typhoid fever (*Typhus abdominalis*), which has occasionally led to confusion in English-language interrogation records and the research literature.

40 Leven, *op. cit.* (note 13), 127–8; William A. Petri , ‘Rickettsiae and Related Organisms’, in Robert S. Porter et al. (eds), The MERCK Manual of Diagnosis and Therapy, 20th edn (Kenilworth, NJ: Merck Sharp & Dohme, 2018), 1691–9, 1694.

41 Leven, *op. cit.* (note 13), 131–3.

42
*Ibid.*, 133; Hubert Fischer , Der deutsche Sanitätsdienst 1921–1945: Organisation, Dokumente und persönliche Erfahrungen, Vol. 4 (Osnabrück: Biblio, 1985), 3444 .

43 Neumann, *Arzttum*, *op. cit.* (note 15), 229.

44 Werther, *op. cit.* (note 15), 121–48; Weindling and Farrell, *op. cit.* (note 12), 106. On typhus epidemics in Auschwitz and their control, see Bogdan Musial , ‘Lagermedizin’ in Auschwitz: Funktion und Dilemmata der Häftlingsärzte 1940–1945 (Hamburg: Hamburger Edition, 2024), 486–95.

45 Hinz-Wessels, *op. cit.* (note 15), 107–12; Weindling and Farrell, *op. cit.* (note 12), 106, 185–7, 198.

46 Hinz-Wessels, *op. cit.* (note 15), 102–3; Weindling and Farrell, *op. cit.* (note 12), 107; Schütz, *op. cit.* (note 16), 147.

47 Werther, *op. cit.* (note 15), 159–60; Hinz-Wessels, *op. cit.* (note 15), 132–3; Weindling, ‘Virologist’, *op. cit.* (note 15), *passim.*

48

Herwig Czech et al., ‘The Lancet Commission on Medicine, Nazism, and the Holocaust: Historical Evidence, Implications for Today, Teaching for Tomorrow’, The Lancet, 402, 10415 (2023), 1867–1940, 188410.1016/S0140-6736(23)01845-737951225.

49

Gerhard Baader , ‘Menschenversuche in Konzentrationslagern’, in Johanna Bleker and Norbert Jachertz (eds), Medizin im ‘Dritten Reich’, 2nd edn (Köln: Deutscher Ärzte-Verlag, 1993), 183–90, 187 .

50

Volker Roelcke , ‘Sulfonamide Experiments on Prisoners in Nazi Concentration Camps: Coherent Scientific Rationality Combined with Complete Disregard of Humanity’, in Sheldon Rubenfeld and Susan Benedict (eds), Human Subjects Research after the Holocaust (Cham: Springer, 2009), 51–66, 62.

51 Czech *et al.*, *op. cit.* (note 48), *passim.*

52 Weindling, *Victims*, *op. cit.* (note 15), 194–7, 223–6 *et passim.* See now the victim database established under the direction of Paul Weindling, which comprises profiles of 30,252 victims of coerced Nazi biomedical research: Deutsche Akademie der Naturforscher Leopoldina – Nationale Akademie der Wissenschaften (ed.), *Victims of Biomedical Research under National Socialism*, June 2025, https://ns-medical-victims.org/ (retrieved 3 February 2026).

53 Leven, *op. cit.* (note 13), 140–3; Neumann, *Arzttum*, *op. cit.* (note 15), 229–32.

54 Browning, *op. cit.* (note 14), 23.

55
*Ibid.*, 24. See also Weindling, *Epidemics*, *op. cit.* (note 15), 273–4; Schütz, *op. cit.* (note 16), 164.

56 Neumann, *Arzttum*, op. cit. (note 15), 232; Süß, *op. cit.* (note 15), 227; Leven, *op. cit.* (note 13), 143–4.

57 Fischer, *op. cit.* (note 42), 3446.

58 Werther, *op. cit.* (note 15), 87–8. See also Neumann, *Arzttum*, *op. cit.* (note 15), 227.

59 Klarsfeld, *op. cit.* (note 30), 127–30, 150–2, 188–9, 252–3; Girstenbrey, *op. cit.* (note 25), 158. See also Leven, *op. cit.* (note 13), 136.

60

Heinrich Mückter , ‘Zur Diagnose des Fleckfiebers’, Münchener Medizinische Wochenschrift, 90, 11 (1943), 195 .

61

Wolfgang Curilla , Der Judenmord in Polen und die deutsche Ordnungspolizei 1939–1945 (Paderborn: Schöningh, 2011), 239–42.

62 Weindling, *Epidemics*, *op. cit.* (note 15), 288.

63

Wolfgang Curilla , Die deutsche Ordnungspolizei und der Holocaust im Baltikum und in Weißrussland 1941–1944, 2nd edn (Paderborn: Schöningh, 2006), 254–6.

64 Stefan Klemp, *‘Nicht ermittelt’: Polizeibataillone und die Nachkriegsjustiz*, 3rd edn (Berlin: Metropol, 2022), 260. The practice of ‘prophylactic’ mass shootings and burnings of actual or potential typhus (and typhoid) carriers is well documented across various regions. See Willi Dreßen and Volker Rieß, ‘Ausbeutung und Vernichtung: Gesundheitspolitik im Generalgouvernement’, in Norbert Frei (ed.), *Medizin und Gesundheitspolitik in der NS-Zeit* (München: Oldenbourg, 1991), 157–71, 166; Dieter Pohl, *Nationalsozialistische Judenverfolgung in Ostgalizien 1941–1944: Organisation und Durchführung eines staatlichen Massenverbrechens*, 2nd edn (München: Oldenbourg, 1997), 149–50, 160–3, 173–4, 241–2, 251, 340; Christian Gerlach, *Kalkulierte Morde: Die deutsche Wirtschafts- und Vernichtungspolitik in Weißrußland 1941 bis 1944* (Hamburg: Hamburger Edition, 1999), 586, 596, 674, 772, 850, 1066, 1069, 1072, 1074, 1095, 1137; Gabriele Lofti, ‘SS-Sonderlager im nationalsozialistischen Terrorsystem: Die Entstehung von Hinzert, Stutthof und Soldau’, in Norbert Frei, Sybille Steinbacher and Bernd C. Wagner (eds), *Ausbeutung, Vernichtung, Öffentlichkeit: Neue Studien zur nationalsozialistischen Lagerpolitik* (München: Saur, 2000), 209–29, 223; Andrej Angrick, *Besatzungspolitik und Massenmord: Die Einsatzgruppe D in der südlichen Sowjetunion 1941–1943* (Hamburg: Hamburger Edition, 2003), 286; Curilla, *op. cit.* (note 63), 160–1, 422, 493, 759; Wendy Lower, *Nazi Empire-Building and the Holocaust in Ukraine* (Chapel Hill, NC: University of North Carolina Press, 2005), 87, 141, 151, 258; Andrej Angrick, ‘Annihilation and Labor: Jews and Thoroughfare IV in Central Ukraine’, in Ray Brandon and Wendy Lower (eds), *The Shoah in Ukraine: History, Testimony, Memorialization* (Bloomington, IN: Indiana University Press, 2008), 190–223, 206, 210–11; Patrick Desbois, *The Holocaust by Bullets: A Priest’s Journey to Uncover the Truth behind the Murder of 1.5 Million Jews* (Basingstoke: Palgrave Macmillan, 2008), 66–7; Yitzhak Arad, *The Holocaust in the Soviet Union* (Lincoln, NE: University of Nebraska Press, 2009), 242–3; Curilla, *op. cit.* (note 61), 285–8, 776.

65 Klarsfeld, *op. cit.* (note 30), 188–9. See also Terrahe’s testimony, *idem*, 252–3.

66
*Ibid.*, 127–30, 150–2.

67
*Ibid.*, 127.

68 Curilla, *op. cit.* (note 61), 321.

69 Schütz, *op. cit.* (note 16), 163–5. See also Weindling, *Epidemics*, *op. cit.* (note 15), 277–8; Curilla, *op. cit.* (note 61), 555; Cain, *op. cit.* (note 15), 246.

70 Hermann Eyer, ‘Die durch Läuse übertragbaren Infektionskrankheiten und ihre Bekämpfung’, *Die medizinische Welt*, 14, 11 (1940), 261–4, 261–2. See also Werther, *op. cit.* (note 15), 72; Schütz, *op. cit.* (note 16), 167.

71 Schütz, *op. cit.* (note 16), 168. See also Klee, *Deutsche Medizin*, *op. cit.* (note 17), 321; Neumann, *Arzttum*, *op. cit.* (note 15), 229.

72 Schütz, *op. cit.* (note 16), 165–6.

73 Browning, *op. cit.* (note 14), *passim*; Dreßen and Rieß, *op. cit.* (note 64), 164–6; Pohl, *op. cit.* (note 64), 120–2, 141, 149–50, 160–3, 173–4, 194, 203, 241–2, 301, 304, 340, 400, 403; Weindling, ‘Die deutsche Wahrnehmung’, *op. cit.* (note 15), 335–9; Gerlach, *op. cit.* (note 64), 103, 596, 674, 689; Bogdan Musial, *Deutsche Zivilverwaltung und Judenverfolgung im Generalgouvernement: Eine Fallstudie zum Distrikt Lublin 1939–1944* (Wiesbaden: Harrassowitz, 1999), 145–6; Weindling, *Epidemics*, *op. cit.* (note 15), 255, 288, 271–7, 426 *et passim*; Caumanns and Esch, *op. cit.* (note 15), *passim*; Friedrich, *op. cit.* (note 15), *passim*; Michael Alberti, *Die Verfolgung und Vernichtung der Juden im Reichsgau Wartheland 1939–1945* (Wiesbaden: Harrassowitz, 2006), 303, 405; Wendy Lower, ‘Anti-Jewish Violence in Western Ukraine, Summer 1941: Varied Histories and Explanations’, in United States Holocaust Memorial Museum (ed.), *The Holocaust in Ukraine: New Sources and Perspectives* (Washington, DC: United States Holocaust Memorial Museum, 2013), 143–73, 153. See also Christopher R. Browning, *The Path to Genocide: Essays on Launching the Final Solution* (Cambridge: Cambridge University Press, 1992). In a meeting on 14 August 1941, Heinrich Himmler (1900–45) explicitly justified the transition to systematic extermination on the grounds that they did not wish ‘to repeat the mistake made in Poland of concentrating the Jews in ghettos. These were merely breeding grounds for epidemics and other diseases’. Martin Cüppers, *Wegbereiter der Shoah: Die Waffen-SS, der Kommandostab Reichsführer SS und die Judenvernichtung 1939–1945* (Darmstadt: WBG, 2005), 183. As late as 1943, when Jews in Eastern Galicia (and elsewhere) were already being shot in large numbers, the district’s propaganda department organised the travelling exhibition *Fleckfieberschau*, which ‘cynically sought to legitimise the mass killings of the alleged “carriers of disease” then taking place’. Pohl, *op. cit.* (note 64), 122.

74 Andrea Hommel and Achim Thom, ‘Verbrecherische Experimente in den Konzentrationslagern’, in Achim Thom and Genadij I. Caregorodcev (eds), *Medizin unterm Hakenkreuz* (Berlin, GDR: Volk und Gesundheit, 1989), 383–400, 387, 395; Fridolf Kudlien, ‘Begingen Wehrmachtsärzte im Rußlandkrieg Verbrechen gegen die Menschlichkeit?’, in Christian Pross and Götz Aly (eds), *Der Wert des Menschen: Medizin in Deutschland 1918–1945* (Berlin: Edition Hentrich, 1989), 333–52; Leven, *op. cit.* (note 13), 138–9; Christian Streit, ‘Das Schicksal der verwundeten sowjetischen Kriegsgefangenen’, in Hannes Heer and Klaus Naumann (eds), *Vernichtungskrieg: Verbrechen der Wehrmacht 1941 bis 1944* (Hamburg: Hamburger Edition, 1995), 78–91, 81–2; Weindling, ‘Die deutsche Wahrnehmung’, *op. cit.* (note 15), 337–8; Weindling, *Epidemics*, *op. cit.* (note 15), 246–59, 361–3; Werther, *op. cit.* (note 15), 160; Neumann, *Arzttum*, *op. cit.* (note 15), 224–5; Neumann, ‘Ausschaltung’, *op. cit.* (note 15), 44–6; Sarah Ehlers, ‘Disease Control and Human Experimentation: Networks, Practices, and Biographical Pathways from Colonial Medicine to Nazi Germany’, in Michelle Gordon and Rachel O’Sullivan (eds), *Colonial Paradigms of Violence: Comparative Analysis of the Holocaust, Genocide, and Mass Killing* (Göttingen: Wallstein, 2022), 83–114.

75 Girstenbrey, *op. cit.* (note 25), 158.

76 Hermann Eyer, ‘Tätigkeitsbericht d. Inst. f. Fleckfieberforschung OKH – Krakau f. d. Zeit vom 1.6. bis 31.7.40’, BArch, RH 12-23/187, ff. 1–6; Eyer, *op. cit.* (note 70), 261; Fischer, *op. cit.* (note 42), 3200; Weindling, *Epidemics*, *op. cit.* (note 15), 334. The first ampoules were delivered in early April 1940. See Leven, *op. cit.* (note 13), 130.

77 Smadel, Schlumberger, and Soles, *op. cit.* (note 31), 3–4; Transcript of the interrogation of Hermann Eyer, 6 November 1946, Josef Daniels to the Dean of the Medical Faculty of the University of Bonn, Erich von Redwitz, 19 November 1946, Ralf Bickhardt to the Dean of the Medical Faculty of the University of Bonn, Erich von Redwitz, 2 December 1946, IPN, GK 164/1736 t. 1, ff. 14–6, 165–8, 253–6; Fischer, *op. cit.* (note 42), 3200–1; Neumann, *Arzttum*, *op. cit.* (note 15), 228.

78 Fischer, *op. cit.* (note 42), 3200–1; Hinz-Wessels, *op. cit.* (note 15), 103.

79 Neumann, *Arzttum*, *op. cit.* (note 15), 229.

80 Institut für Fleckfieber- und Virusforschung des OKH, appointments of officers and officials in officer rank, 1 February 1945, BArch, RH 15/197, fo. 122; Smadel, Schlumberger, and Soles, *op. cit.* (note 31), 3–4; Heinrich Mückter to the Dean of the Medical Faculty of the University of Bonn, Erich von Redwitz, 19 November 1946, IPN, GK 164/1736 t. 1, ff. 217–21; Curriculum vitae of Heinrich Mückter, undated (after 1961), LAV NRW R, Gerichte Rep. 139 Nr. 188a, fo. 17. See also Fischer, *op. cit.* (note 42), 3201; Schütz, *op. cit.* (note 16), 151.

81 For Rudolf Weigl, see Allen, *op. cit.* (note 15), *passim.*

82
*Ibid.*, 92–5, 106–7; Weindling, *Epidemics*, *op. cit.* (note 15), 231, 335; Hinz-Wessels, *op. cit.* (note 15), 103; Schütz, *op. cit.* (note 16), 146.

83 The titles were: ‘Über die rickettsicide Wirksamkeit der gebräuchlichen Entwesungsmittel’ [On the Rickettsicidal Efficacy of Commonly Used Vermin Control Agents], ‘Über die rickettsicide Wirksamkeit der gebräuchlichen chemischen Entwesungsmittel’ [On the Rickettsicidal Efficacy of Commonly Used Chemical Vermin Control Agents], ‘Die wertmäßige Beurteilung von Fleckfieber-Impfstoffen’ [The Value Assessment of Typhus Vaccines], ‘Ein Verfahren zur Sterilisation sporenhaltiger Impfstoffe’ [A Process for the Sterilisation of Spore-Containing Vaccines]. See Smadel, Schlumberger, and Soles, *op. cit.* (note 31), 15–6.

84 Schütz, *op. cit.* (note 16), 152.

85 J.B. Rice, *Tropical Medicines and Other Medical Subjects in Germany* (London: Combined Intelligence Objectives Sub-Committee, 1945), 15; Weindling, *Epidemics*, *op. cit.* (note 15), 350–1.

86 Affidavit of Hermann Eyer, undated (1946), IPN, GK 164/1736 t. 1, ff. 231–9.

87 Forschungsgruppe der Militärärztlichen Akademie, *Kampf dem Fleckfieber!*, produced by Heeres-Filmstelle, 1941, BArch, Film 4273, min. 18:34–32:45. The greater part of the film can be attributed to pathologist Paul Schürmann (1895–1941), who served as commander of the Berlin Military Medical Academy’s research group (*Lehrgruppe C*). The footage was filmed by Schürmann and his film unit between 17 and 26 March 1941 in occupied Poland but was presumably post-produced without Schürmann’s involvement. By May, he had already departed for another film project in Crete and was killed during his final deployment in Belarus on 2 July 1941. *Kampf dem Fleckfieber!* premiered in Berlin in December 1941. Its antisemitic opening scenes (especially min. 1:11–2:55) can presumably be attributed to Hermann Eyer. These scenes stand out markedly from the otherwise largely sober and factual tone of the documentary. See Volker Hartmann, ‘Oberstarzt Professor Dr. Paul Schürmann (1895–1941), Namensgeber des Preises der Deutschen Gesellschaft für Wehrmedizin und Wehrpharmazie e.V.: Eine kritische Analyse’, *Wehrmedizin und Wehrpharmazie*, 69, 3 (2025), 62–79, 73–5. For the dating of the footage, see also min. 24:36 (showing a date stamp of 21 March 1941); for the premiere, see Anonymous, ‘Tagesgeschichtliche Notizen’, *Münchener Medizinische Wochenschrift*, 89, 3 (1942), 69–70, 70. Hermann Eyer can be clearly identified in several scenes at the Krakow institute (min. 18:07–14, 19:55–20:03, 28:20–29:37, 32:16–22). A contemporary comparison photograph of Eyer can be found in Allen, *op. cit.* (note 15), 92. See also Klee, *Deutsche Medizin*, *op. cit.* (note 17), 320. By contrast, the identification of a physician (min. 6:51–7:05, 8:44–9:00) as Heinrich Mückter – allegedly conducting human experiments in the Krakow laboratories – as claimed by Nuding, *op. cit.* (note 4), 18, 40–1, is entirely unfounded. Mückter was still stationed in Norway until April 1941. Moreover, the scenes in question were clearly not filmed at the Krakow institute (which appears only in the second half of the film, min. 17:24–32:45), but rather at a military infirmary. They depict an unknown Wehrmacht physician examining German soldiers. A comparison with a contemporary photograph of Mückter rules out any identification. See the photo attached to Mückter’s DFG questionnaire dated 24 October 1939, BArch, R 73/13252.

88 Rice, *op. cit.* (note 85), 18–9; Leven, *op. cit.* (note 13), 129–30; Allen, *op. cit.* (note 15), 145–7. For details, see Stefan Kryński , Eugeniusz Becla and Marian Machel , ‘Weigl’s Method of Intrarectal Inoculation of Lice in Production of Typhus Vaccine and Experimental Works with Rickettsia Prowazeki’, Annales Academiae Medicae Gedanensis, 4 (1974), 19–51 ; Waclaw Szybalski , ‘Maintenance of Human-Fed Live Lice in the Laboratory and Production of Weigl’s Exanthematous Typhus Vaccine’, in Karl Maramorosch and Farida Mahmood (eds), Maintenance of Human, Animal, and Plant Pathogen Vectors (Enfield, NH: Science Publishers, 1999), 159–80.

89 Werther, *op. cit.* (note 15), 199.

90 Weindling, *Epidemics*, *op. cit.* (note 15), 350. See also Cain, *op. cit.* (note 15), 258, 263.

91 Lawyer Alphons M. Kugelmeier, Bonn, to Military Government, War Crimes Investigation Unit, Oeynhausen, 14 May 1947, Heinrich Mückter to the Dean of the Medical Faculty of the University of Bonn, Erich von Redwitz, 19 November 1946, Affidavit of Hermann Eyer, undated (1946), IPN, GK 164/1736 t. 1, ff. 50–3, 217–21, 231–9. See also Weindling, *Epidemics*, *op. cit.* (note 15), 351–2.

92 Smadel, Schlumberger, and Soles, *op. cit.* (note 31), 6.

93 Schütz, *op. cit.* (note 16), 152, 171–2. See also Heinrich Mückter to the Dean of the Medical Faculty of the University of Bonn, Erich von Redwitz, 19 November 1946, IPN, GK 164/1736 t. 1, ff. 217–21.

94 Schütz, *op. cit.* (note 16), 148–9. See also IPN, GK 164/1736 t. 1. For Josef Daniels and his work at the OKH Institute, see Smadel, Schlumberger, and Soles, *op. cit.* (note 31), 5; Josef Daniels to the Dean of the Medical Faculty of the University of Bonn, Erich von Redwitz, 19 November 1946, IPN, GK 164/1736 t. 1, ff. 165–8; Allen, *op. cit.* (note 15), 165. Daniels later served as president of the Federal Health Office (*Bundesgesundheitsamt*) from 1964 to 1969. See Schütz, *op. cit.* (note 16), 151.

95 Original English translation of the testimony by Zdzisław Przybyłkiewicz on the action sheet concerning Heinrich Mückter, 25 July 1947, IPN, GK 164/1736 t. 3, fo. 13.

96 Schütz, *op. cit.* (note 16), 150–1. See also Allen, *op. cit.* (note 15), 276–9.

97 Schütz, *op. cit.* (note 16), 158–62, 172.

98 Heinrich Mückter, ‘Vorläufiger Bericht über Quintana-Versuche’, 15 November 1944, Heinrich Mückter, ‘Versuche zur Quintana-Prophylaxe u. -Therapie’ and covering letter to Hermann Eyer, 30 December 1944, IPN, GK 164/1736 t. 1, ff. 101–24. See also Martin Johnson , Raymond G. Stokes and Tobias Arndt , The Thalidomide Catastrophe (Exeter: Onwards and Upwards, 2018), 60–2, which includes the full citation of a report by a louse-feeder who was used as a test subject in the first series of trials (IPN, MBr 2837/01/07). The trench fever vaccine trials and their negative outcome are mentioned without further specification in Hermann Eyer , ‘Allgemeines über Rickettsiosen’, in Gustav von Bergmann , Walter Frey and Herbert Schwiegk (eds), Handbuch der Inneren Medizin, 4th edn, Vol. 1/1 (Berlin/Heidelberg: Springer, 1952), 638–82, 644, 657. See also Rice, *op. cit.* (note 85), 16.

99 Smadel, Schlumberger, and Soles, *op. cit.* (note 31), 9; Richard Bieling, ‘Fünftagefieber, Läusefieber, kurz dauerndes Fieber’, *Deutsche Medizinische Wochenschrift*, 72, 33–4 (1947), 479–81; Eyer, *op. cit.* (note 98), 644; Werner Mohr and Fritz Weyer , ‘Wolhynisches Fieber’, in Otto Gsell and Werner Mohr (eds), Infektionskrankheiten, Vol. 4 (Berlin/Heidelberg: Springer, 1972), 117–32; Weindling, *Epidemics*, *op. cit.* (note 15), 89–90; Larry M. Bush and Maria T. Vazquez-Pertejo , ‘Gram-Negative Bacili’, in Robert S. Porter et al. (eds), The MERCK Manual of Diagnosis and Therapy, 20th edn (Kenilworth, NJ: Merck Sharp & Dohme, 2018), 1577–99, 1579 .

100 Heinrich Mückter, ‘Vorläufiger Bericht über Quintana-Versuche’, 15 November 1944, IPN, GK 164/1736 t. 1, ff. 103–10. A former louse-feeder, who had unwittingly been used as an experimental subject, recalled: ‘Shortly after adoption [as louse-feeders] we found out that we would receive some injections, which were supposed to protect from the trench fever […]. After I started my work at the laboratory I found out that the injection I received earlier wasn’t an inoculation, but I was injected with very virulent germs of trench fever’. Johnson, Stokes, and Arndt, *op. cit.* (note 98), 60–2.

101 Schütz, *op. cit.* (note 16), 159.

102 Heinrich Mückter, ‘Vorläufiger Bericht über Quintana-Versuche’, 15 November 1944, IPN, GK 164/1736 t. 1, ff. 103–10, quoted from fo. 108.

103 Heinrich Mückter, ‘Versuche zur Quintana-Prophylaxe u. -Therapie’, 30 December 1944, IPN, GK 164/1736 t. 1, ff. 111–24, quoted from fo. 122. See also Johnson, Stokes, and Arndt, *op. cit.* (note 98), 61–2: ‘The illness of mine was very hard: in evening time a high fever – 41.3 Celsius. I remember it as the highest fever I’ve ever had. At the morning about 35 Celsius, this variation made me feel extremely exhausted. Apart from that, of course, headache and the worst ever ache of shin-bone. […] From what I found out […] few people were seriously harmed because the illness caused damage to their kidneys, and others were diagnosed with the meningitis’. Trench fever often progressed with such severe pain that Wehrmacht physicians experimented with symptomatic treatment using opiates. See Feldlazarett (mot) 680, Kranken-Abteilung I, Proposal for the symptomatic treatment of trench fever using opiates, 2 May 1943, BArch, RH 12-23/357.

104

Richard Bieling and Lilly Oelrichs , ‘Experimentelle Untersuchungen über die Infektion mit Rickettsia quintana (pediculi)’, Zeitschrift für Hygiene und Infektionskrankheiten, 127 (1947), 41–820262129. For the animal model of human disease and its application in research, see Volker Roelcke , ‘The Animal Model of Human Disease as a Core Concept of Medical Research: Historical Cases, Failures, and Some Epistemological Considerations’, Science in Context, 35, 2 (2022), 173–9738084861 10.1017/S0269889723000170; Volker Roelcke , ‘Das Tiermodell menschlicher Krankheit: Zur Geschichte der epistemologischen und ethischen Dimension eines zentralen Konzepts der medizinischen Krankheitsforschung’, in Johann S. Ach (ed.), Tiere in der Medizin (Paderborn: Brill mentis, 2024), 87–107 .

105

Jochen Vollmann and Rolf Winau , ‘Informed Consent in Human Experimentation before the Nuremberg Code’, The British Medical Journal, 313, 7070 (1996), 1445–98973233 10.1136/bmj.313.7070.1445PMC2352998; Hinz-Wessels, *op. cit.* (note 15), 128–31; Volker Roelcke , ‘The Use and Abuse of Medical Research Ethics: The German “Richtlinien”/Guidelines for Human Subject Research as an Instrument for the Protection of Research Subjects – and of Medical Science, ca. 1931–1961/64’, in Paul Weindling (ed.), From Clinic to Concentration Camp: Reassessing Nazi Medical and Racial Research, 1933–1945 (London: Routledge, 2017), 33–56 .

106

Reichsministerium des Innern , ‘Endgültiger Entwurf von Richtlinien für neuartige Heilbehandlung und für die Vornahme wissenschaftlicher Versuche am Menschen’, Klinische Wochenschrift, 10, 13 (1931), 623 .

107 Weindling and Farrell, *op. cit.* (note 12), 124–5. This, however, was due to the belief that typhus might manifest in adolescents in a manner similar to a childhood illness. See Erwin Ding, ‘Versuchsreihe über Fleckfieberimpfstoff nach Wohlrab und Gildemeister-Reiter’, 2 January 1942, Landesarchiv Thüringen – Hauptstaatsarchiv Weimar (Thuringian State Archives – Main State Archive Weimar, hereafter abbreviated LATh – HStA Weimar), Konzentrationslager und Haftanstalten Buchenwald Nr. 62, fo. 1b.

108 Reichsministerium des Innern, *op. cit.* (note 106), 623.

109

Rudolf Weigl , ‘Immunization against Typhus Fever in Poland during World War II’, Texas Reports on Biology and Medicine, 5, 2 (1947), 177–9, 177 20255936 . See also Rudolf Weigl , ‘Metody walki z durem osutkowym’, Przegląd Epidemiologiczny, 2, 1–2 (1948), 3–19, 16–9. For details of the 1942–4 study of 7,830 vaccinated persons (527 infected), see Henryk Mosing, ‘Tyfus plamisty u osób szczepionych’, *Rozprawy Wydziału Lekarskiego PAU*, 8, 4 (1948), 93–125. Since typhus infections among louse-feeders regularly occurred as part of the normal production process of the Weigl vaccine, louse-feeders were systematically vaccinated with different vaccines and at varying dosage levels and subsequently, according to Mosing, *idem*, 96, ‘infected by voluntarily feeding infected lice’. See also Cain, *op. cit.* (note 15), 261–2. For a similar practice in Warsaw, see Rudolf Wohlrab , ‘Prüfung von Fleckfieberimpfstoffen durch Feststellung des Agglutinintiters’, Schriftenreihe für Seuchenbekämpfung, 1 (1944), 42–9. For the rationale in Warsaw, see Rudolf Wohlrab , ‘Immunität und Schutzimpfungsverfahren bei den Erkrankungen der Fleckfiebergruppe’, Schriftenreihe für Seuchenbekämpfung, 1 (1944), 22–41, 37: ‘As we lack experimental animals, particularly guinea pigs, […] the development of up-to-date testing methods is urgently required. One possible approach would be to determine the agglutinin titres produced by the vaccines, first in experimental animals and then in humans’. For the method of determining agglutination titres (‘Weigl’s reaction’), see also Hermann Eyer and Hermann Dillenberg , ‘Die Serodiagnostik des Fleckfiebers: Eine vergleichende Untersuchung’, Zeitschrift für Hygiene und Infektionskrankheiten, 125 (1943), 308–30; Henryk Mosing , ‘Methods of Evaluation of Typhus Vaccine Potency’, Texas Reports on Biology and Medicine, 5, 2 (1947), 173–620255935 .

110 Anonymous, ‘Typhus Vaccines: A Crucial Experiment’, *The Lancet*, 242, 6277 (1943), 770.

111

Erwin Ding , ‘Über die Schutzwirkung verschiedener Fleckfieberimpfstoffe beim Menschen und den Fleckfieberverlauf nach Schutzimpfung’, Zeitschrift für Hygiene und Infektionskrankheiten, 124 (1943), 670–82.

112 In fact, the papers published under Ding’s name were primarily written by Ding’s clerk Eugen Kogon and the Polish inmate physician Marian Ciepielowski (1907–73). See Kogon, *op. cit.* (note 11), 246. For the various scientific articles published under Ding’s name between 1942 and 1945, see Eva Hedfors , ‘Medical Science in the Light of the Holocaust: Departing from a Post-War Paper by Ludwik Fleck’, Social Studies of Science, 38, 2 (2008), 259–83, 267–7318831133 10.1177/0306312707082953; Allen, *op. cit.* (note 15), 208, 342.

113 Anonymous, *op. cit.* (note 110), 770.

114 Weindling, *Epidemics*, *op. cit.* (note 15), 356.

115
*Ibid.*, 353–4; Allen, *op. cit.* (note 15), 79–83, 99, 103–5, 195, 200–1, 233; Weindling, *Victims*, *op. cit.* (note 15), 94–5; Weindling and Farrell, *op. cit.* (note 12), 111–15, 190–2. For the SS Medical Academy, see Ines Hopfer , ‘Die Spur führt nach Graz: Auf der Suche nach den sterblichen Überresten eines NS-Opfers’, Jahrbuch der KZ-Gedenkstätte Mauthausen, 2 (2008), 48–57 ; Florian Bruns , Medizinethik im Nationalsozialismus: Entwicklungen und Protagonisten in Berlin (1939–1945) (Stuttgart: Steiner, 2009), 69 . For Joachim Mrugowsky and the SS Hygiene Institute, see *idem*, 131–66; Weindling, *Epidemics*, *op. cit.* (note 15), 250–9, 352–7 *et passim*; Weindling, *Fleckfieberforschung*, *op. cit.* (note 15), *passim.*

116 Although he himself did not use the combined form of the surname, the double-barreled name ‘Ding-Schuler’ became established after 1945.

117 Weindling and Farrell, *op. cit.* (note 12), 115–25.

118
*Ibid.*, 118.

119
*Ibid.*, 124. See also Schneider and Stein, *op. cit.* (note 15), 30–2.

120 Klaus Dörner, Angelika Ebbinghaus and Karsten Linne (eds), *Der Nürnberger Ärzteprozeß 1946/47*: *Wortprotokolle, Anklage- und Verteidigungsmaterial, Quellen zum Umfeld*, microfiche edn (München: Saur, 1999), 2/01204, 2/01247–8, quoted from 2/01247. See also Kogon, *op. cit.* (note 11), 136.

121 Weindling and Farrell, *op. cit.* (note 12), 118–21, 170–4, 182–7. See also Schneider and Stein, *op. cit.* (note 15), 23–7; Baader, *op. cit.* (note 49), 187; Werther, *op. cit.* (note 15), 45; Hinz-Wessels, *op. cit.* (note 15), 102–13. That the Weigl vaccine was not the primary object of investigation and served merely as a benchmark is also evidenced by the original trial design of Trial Series I, which stipulated that only the new vaccines developed by the Robert Koch Institute and the Behringwerke were to be tested. The series was only expanded at short notice to include the OKH Weigl vaccine following a telephone order from Ding-Schuler to the SS camp physician Waldemar Hoven (1903–48). It is even possible that the local stock of the OKH vaccine – originally intended for immunising SS personnel and the prisoner-functionaries at the experimental station – was spontaneously drawn upon for this purpose. See Erwin Ding, ‘Versuchsreihe über Fleckfieberimpfstoff nach Wohlrab und Gildemeister-Reiter’, 2 January 1942, Lagerarzt KL Buchenwald, Waldemar Hoven, to Lagerkommandant KL Buchenwald, 9 January 1942, LATh – HStA Weimar, Konzentrationslager und Haftanstalten Buchenwald Nr. 62, ff. 1a–b. For Waldemar Hoven, see Ryan A. Farrell, ‘“The Lowest Grade as a Human Being and Physician”: The Relationships, Corruption and Power of Waldemar Hoven at Buchenwald Concentration Camp, 1939–1943’ (MA thesis: Oxford Brookes University, 2013); Marco Pukrop , ‘ *SS-Mediziner zwischen Lagerdienst und Fronteinsatz: Die personelle Besetzung der medizinischen Abteilung im Konzentrationslager Sachsenhausen* ’ (PhD thesis: University of Hannover, 2015), 99–100, 190, 601–2; Weindling, *Victims*, *op. cit.* (note 15), 62, 95–99, 183, 202, 205–7; Herlinde Pauer-Studer and J. David Velleman , *‘* Weil ich nun mal ein Gerechtigkeitsfanatiker bin’: Der Fall des SS-Richters Konrad Morgen (Berlin: Suhrkamp, 2017), 142, 156–78, 304 *et passim* ; Heiko Wegmann, ‘Waldemar Hoven: Eine Melodie vor sich hinpfeifend – Der Lagerarzt des KZ Buchenwald’, in Wolfgang Proske (ed.), *Täter, Helfer, Trittbrettfahrer: NS-Belastete aus Baden-Württemberg*, Vol. 6 (Gerstetten: Kugelberg-Verlag, 2017), 176–89; Steger and Jeskow, *op. cit.* (note 34), 146–8.

122 Reichsarzt SS und Polizei – Leitender Hygieniker, Berlin, Joachim Mrugowsky, to Fleckfieber-Forschungsinstitut OKH, Krakau, Hermann Eyer, *et al.*, 5 May 1942, BArch, NS 33/343; Dörner, Ebbinghaus and Linne, *op. cit.* (note 120), 2/01206, 2/01247–8, 4/05056–8; Neumann, *Arzttum*, *op. cit.* (note 15), 231; Schütz, *op. cit.* (note 16), 156–8. In Ding’s aforementioned journal article, this version was modified to state that the infections had occurred during the three winters from 1940 to 1943 among physicians, nursing staff, soldiers, and civilian personnel deployed in areas and assignments ‘in which contact with typhus and lice was particularly close and, with a high degree of probability, led to illness’. Ding, *op. cit.* (note 111), 670.

123 Rijksarchief in België, Dienst Archief Oorlogsslachtoffers (State Archives of Belgium, Archives Service for War Victims, hereafter abbreviated AOS [until 2017: Federale Overheidsdienst Sociale Zekerheid, Directie-generaal Oorlogsslachtoffers – Dienst voor de Oorlogsslachtoffers, Dienst Archief en Documentatie]), R 696/Tr 255612/1542–8. In addition to private and official correspondence, parts of the original department’s diary (‘Ding diary’, 3 December 1942 to 30 June 1944), the correspondence log (12 October 1943 to 29 March 1945), the record of postal dispatches (25 March 1944 to 5 March 1945), and postal receipts (9 March 1944 to 26 March 1945) have survived and been thoroughly examined. Transcriptions of both the already known Ding diary – retrospectively compiled by Eugen Kogon and Ding-Schuler immediately before the camp’s liberation and later used in the Nuremberg Doctors’ Trial – and of the original version discovered in Brussels can be found in Weindling and Farrell, *op. cit.* (note 12), 170–213. For the provenance, see *idem*, 115–7, 125–9, 170, 190, 199; Allen, *op. cit.* (note 15), 319; Jens-Christian Wagner, ‘Selektion und Segregation: Vernichtung und Arbeit am Beispiel Mittelbau-Dora’, in Marc Buggeln and Michael Wildt (eds), *Arbeit im Nationalsozialismus* (München: De Gruyter Oldenbourg, 2014), 329–47, 333.

124 Erwin Ding, ‘Tagebuch der Abteilung für Fleckfieber- und Virusforschung Weimar-Buchenwald am Hygiene-Institut der Waffen-SS für 1944’, 5 January to 30 June 1944, AOS, R 696/Tr 255612/1547; Weindling and Farrell, *op. cit.* (note 12), 206.

125 Hygiene-Institut der Waffen-SS, Abteilung für Fleckfieber- und Virusforschung Weimar-Buchenwald, Erwin Ding, to Hygiene-Institut der Waffen-SS Berlin, Joachim Mrugowsky, 4 March 1944, AOS, R 696/Tr 255612/1548, fo. 169. See also Allen, *op. cit.* (note 15), 246. The provision of this material may have gone back to the initiative of Richard Bieling (1888–1967), head of the Serobacteriological Department of the Behringwerke in Marburg and Consulting Hygienist of the Wehrmacht, who maintained regular contact with Mrugowsky and Ding-Schuler. In the summer of 1943, Bieling had discussed with Eyer the plan to establish at the Krakow institute a collection and distribution centre for rickettsial strains ‘which could then be made available to the various institutes charged with producing the vaccine for army purposes’. Beratender Hygieniker Außenstelle Süd, Richard Bieling, Experience report, August 1943, BArch, RH 12-23/187, ff. 181–3. For Bieling, see Klee, *op. cit.* (note 10), 48–9; Klee, *Auschwitz*, *op. cit.* (note 17), 299, 310, 319; Weindling, *Epidemics*, *op. cit.* (note 15), 347–9, 354, 362; Weindling, *Victims*, *op. cit.* (note 15), 50, 101. In December 1944, the first 11,000 doses of the SS’s own vaccine, in the version intended for prisoners (the so-called *Rotpunkt-Impfstoff*), were delivered to the Buchenwald camp pharmacy. See Madeleine Ritters , Dominik Groß and Nico Biermanns , ‘Drug Shortage and Networks of Medical Crime: Herbert Siggelkow (1906–1976), Chief Pharmacist of the Nazi Concentration Camps’, Die Pharmazie, 80, 11–12 (2025), 175–87, 18310.1691/ph.2025.507641491388. See also Hackett, *op. cit.* (note 11), 73–4; Allen, *op. cit.* (note 15), 247.

126 Telegram Hygiene-Institut der Waffen-SS, Abteilung für Fleckfieber- und Virusforschung Weimar-Buchenwald, Erwin Ding, to Oberster Hygieniker, Joachim Mrugowsky (via Sanitätsamt der Waffen-SS), 10 July 1944, AOS, R 696/Tr 255612/1548, fo. 86.

127 Weindling, *Epidemics*, *op. cit.* (note 15), 333; Weindling, *Victims*, *op. cit.* (note 15), 98–9; Weindling and Farrell, *op. cit.* (note 12), 115.

128 Hermann Eyer, ‘Über das Fleckfieber und seine neuzeitliche Bekämpfung’, *Wiener Klinische Wochenschrift*, 56, 45–6 (1943), 657–61, 660; Smadel, Schlumberger, and Soles, *op. cit.* (note 31), 3–9, 13; Leven, *op. cit.* (note 13), 142; Schütz, *op. cit.* (note 16), 158. Ralf Bickhardt had previously collaborated with Richard Otto (1872–1952) at the State Institute for Experimental Therapy in Frankfurt am Main on novel vaccine production methods using mouse lungs and egg yolks, which he subsequently introduced at the OKH institute. Bickhardt was released from British captivity in October 1945 and subsequently worked at the Schering plant in Bückeburg, Lower Saxony. In the 1960s, he served as head of the clinical and serological laboratory at the University Dermatology Clinic in Freiburg im Breisgau. See Ralf Bickhardt, ‘Über Fleckfieber-Schutzimpfstoffe aus den Frankfurter Instituten’, *Arbeiten aus dem Staatlichen Institut für experimentelle Therapie und dem Forschungsinstitut für Chemotherapie zu Frankfurt a.M.*, 42 (1943), 12–16; Richard Otto, ‘Bericht über die Tätigkeit des Forschungsinstituts für Chemotherapie zu Frankfurt a.M. in der Zeit vom März 1939 bis Ende März 1943’, *Arbeiten aus dem Staatlichen Institut für experimentelle Therapie und dem Forschungsinstitut für Chemotherapie zu Frankfurt a.M.*, 43 (1943), 32–48; Ralf Bickhardt to the Dean of the Medical Faculty of the University of Bonn, Erich von Redwitz, 2 December 1946, IPN, GK 164/1736 t. 1, ff. 14–6; Richard Otto, *Bericht über die Tätigkeit des Staatl. Instituts für experimentelle Therapie in Frankfurt a.M. in der Zeit vom 1. April 1941 bis 31. Dezember 1943* (Wiesbaden: Dieterich, 1947), 17–9; Albert-Ludwigs-Universität Freiburg im Breisgau (ed.), *Personen- und Vorlesungsverzeichnis Sommersemester 1965* (Freiburg im Breisgau: Goldschagg, 1965), 81.

129 Weindling and Farrell, *op. cit.* (note 12), 116, 171–3.

130

Rudolf Wohlrab , ‘Immunisierungen gegen Flecktyphus’, Medizinische Klinik, 37, 21 (1941), 532–5; Richard Otto and Ralf Bickhardt , ‘Weitere experimentelle Untersuchungen über Schutzimpfstoffe gegen Flecktyphus: Impfstoffe aus rickettsienhaltigen Mäuselungen’, Zeitschrift für Hygiene und Infektionskrankheiten, 123 (1942), 717–24.

131 Reichsarzt SS und Polizei – Leitender Hygieniker, Berlin, Joachim Mrugowsky, to Fleckfieber-Forschungsinstitut OKH, Krakau, Hermann Eyer, *et al.*, 5 May 1942, BArch, NS 33/343; [Waldemar Hoven], ‘Zwischenbericht zum Impfstoffversuch “Paris” und “Bukarest”’, 10 November 1942, LATh – HStA Weimar, Konzentrationslager und Haftanstalten Buchenwald Nr. 62, ff. 22–3; Ding, *op. cit.* (note 111); Anonymous, *op. cit.* (note 110); Kenneth Mellanby, ‘Medical Experiments on Human Beings in Concentration Camps in Nazi Germany’, *The British Medical Journal*, 1 [*sic!*], 4490 (1947), 148–50, 150; Baader, *op. cit.* (note 49), 187. On Eyer’s shift in attitude, see Eyer, *op. cit.* (note 128), 660; Hermann Eyer , ‘Über Fragen der neuzeitlichen Fleckfieberbekämpfung’, Münchener Medizinische Wochenschrift, 90, 38–9 (1943), 569; Hermann Eyer , ‘ *Fleckfieber* ’, in Siegfried Handloser and Wilhelm Hoffmann (eds), *Wehrhygiene* (Berlin: Springer, 1944), 49–57, 56 ; Schütz, *op. cit.* (note 16), 157–8. The results of the first two trial series formed the basis of Ding’s article published in June 1943. Perhaps because Trial Series III–VII failed, Weindling and Farrell, *op. cit.* (note 12), 118–9, mistakenly assume that the first two trial series had also failed. The fact that Eyer – and, with him, much of contemporary bacteriological research – evidently regarded the research findings from Buchenwald as reliable is reflected in the chapter on rickettsioses he authored for the 1952 *Handbuch der Inneren Medizin*, in which he cited no fewer than four different articles by Ding/Schuler. See Eyer, *op. cit.* (note 98), 665–6, 670, 672. See also the references to Ding’s 1943 article in Joseph F. Sadusk Jr., ‘Typhus Fever in the United States Army Following Immunization’, Journal of the American Medical Association, 133, 16 (1947), 1192–920291495 10.1001/jama.1947.02880160018004; John C. Snyder , ‘The Typhus Fevers’, in Thomas M. Rivers (ed.), Viral and Rickettsial Infections of Man (Philadelphia, PA: Lippincott, 1948), 462–92; Herald R. Cox , ‘Viral Vaccines and Human Welfare’, The Lancet, 262, 6775 (1953), 1–5 10.1016/s0140-6736(53)90001-413053762; Walther Schäfer , ‘Die Fleckfieberschutzimpfung’, in Albert Herrlich (ed.), Handbuch der Schutzimpfungen (Berlin/Heidelberg: Springer, 1965), 634–40; Larry J. Wright et al., ‘Laboratory-Acquired Typhus Fevers’, Annals of Internal Medicine, 69, 4 (1968), 731–85682246 10.7326/0003-4819-69-4-731.

132 Weigl, ‘Immunization’, *op. cit.* (note 109), 177; Mosing, ‘Tyfus’, *op. cit.* (note 109), 96–7, 118–9, 123–5; Schütz, *op. cit.* (note 16), 158–62.

133 Schütz, *op. cit.* (note 16), 145, 153, 155–7, 162.

134 Klee, *Deutsche Medizin*, *op. cit.* (note 17), 320–1; Klee, *op. cit.* (note 10), 142.

135 Weindling and Farrell, *op. cit.* (note 12), 119–20.

136 Klee, *Auschwitz*, *op. cit.* (note 17), 345. See also Neumann, *Arzttum*, *op. cit.* (note 15), 232.

137 Werner Scherf, ‘Die Verbrechen der SS-Ärzte im KZ Buchenwald – der antifaschistische Widerstand im Häftlingskrankenbau, 2. Beitrag: Juristische Probleme’ (LLD thesis: Humboldt University of Berlin, 1987), 146–53, 394; Schneider and Stein, *op. cit.* (note 15), 26, 36–7; Klee, *Auschwitz*, *op. cit.* (note 17), 328–9; Dörner, Ebbinghaus and Linne, *op. cit.* (note 120), 4/5432–3; Hinz-Wessels, *op. cit.* (note 15), 58; Weindling and Farrell, *op. cit.* (note 12), 176; Standortarzt der Waffen-SS Weimar, Waldemar Hoven, to Chef des Amtes D III Oranienburg, Enno Lolling, 15 January 1943, LATh – HStA Weimar, Konzentrationslager und Haftanstalten Buchenwald Nr. 62, ff. 27–8. For Enno Lolling (1888–1945), see Pukrop, *op. cit.* (note 121), 30, 75–7, 82–5, 98–101, 604–5 *et passim.*

138 Scherf, *op. cit.* (note 137), 73, 147; Klee, *Auschwitz*, *op. cit.* (note 17), 329; Dörner, Ebbinghaus and Linne, *op. cit.* (note 120), 2/010956–7; Weindling and Farrell, *op. cit.* (note 12), 177; Schütz, *op. cit.* (note 16), 155. For Bernhard Schmidt, see Scherf, *op. cit.* (note 137), 333–4; Klee, *Auschwitz*, *op. cit.* (note 17), 343–5; Neumann, *Arzttum*, *op. cit.* (note 15), 374; Klee, *op. cit.* (note 10), 544.

139 Scherf, *op. cit.* (note 137), 73, 149; Klee, *Auschwitz*, *op. cit.* (note 17), 343–4; Dörner, Ebbinghaus and Linne, *op. cit.* (note 120), 2/010422–3, 2/010956–7, 4/04111; Schütz, *op. cit.* (note 16), 162.

140 Robert-Koch-Institut Berlin, Martin Krüpe, to Standortarzt der Waffen-SS Weimar, Waldemar Hoven, 9 February 1943, LATh – HStA Weimar, Konzentrationslager und Haftanstalten Buchenwald Nr. 62, fo. 31: ‘The hollow rod with a ring [*Hohlstab mit Ring*] represents a considerable improvement for opening the ampoules. We intend to include this hollow rod as part of the vaccination equipment’. For Martin Krüpe (1910–81), see Scherf, *op. cit.* (note 137), 151, 327; Klee, *Auschwitz*, *op. cit.* (note 17), 134–5; Klee, *op. cit.* (note 10), 344–5; Hinz-Wessels, *op. cit.* (note 15), 57, 70.

141 Schneider and Stein, *op. cit.* (note 15), 37; Klee, *Auschwitz*, *op. cit.* (note 17), 329; Weindling and Farrell, *op. cit.* (note 12), 176. For the yellow fever vaccine produced in Krakow, see Smadel, Schlumberger, and Soles, *op. cit.* (note 31), 8. There is evidence that the written reports on these tolerability trials were sent to Eyer directly from Buchenwald. See Scherf, *op. cit.* (note 137), 151, 394.

142 Lagerarzt KL Buchenwald, Heinrich Plaza, to Erwin Ding, 27 February 1943, LATh – HStA Weimar, Konzentrationslager und Haftanstalten Buchenwald Nr. 62, fo. 34: ‘The cause of death determined at autopsy by Dr [Otto] Hübner of the Pathological Institute in Jena was: “General sepsis with abscess-forming pleuritis”. Thus, there was no causal link between the yellow fever vaccination and the cause of death’. Scherf, *op. cit.* (note 137), 394, comments: ‘Plaza’s interpretation is premature; a definitive assessment would only be possible after examining the liver sections announced in the letter. In fact, the reported incubation period could indicate a connection to the vaccination’. For Heinrich Plaza (1912–68), see *idem*, 299–302, 353; Nico Biermanns and Mathias Schmidt, ‘Aufgabenbereiche, Handlungsspielräume und Nachkriegsnarrative eines SS-Lagerarztes: August Bender (1909–2005) im KZ Buchenwald und in der Bundesrepublik’, *Medizinhistorisches Journal*, 57, 4 (2022), 363–415, 375–7. For Otto Hübner (b. 1909), see Scherf, *op. cit.* (note 137), 324, 347; Susanne Zimmermann, *Die Medizinische Fakultät der Universität Jena während der Zeit des Nationalsozialismus* (Berlin: Verlag für Wissenschaft und Bildung, 2000), 175–8.

143 Scherf, *op. cit.* (note 137), 76–7; Klee, *Auschwitz*, *op. cit.* (note 17), 343.

144 Weindling and Farrell, *op. cit.* (note 12), 116, 119, 174–6.

145

Mathias Schütz , ‘Memory Unbound, Unfounded Memory? On Medicine, the Holocaust, and Bioethical Reflection’, European Journal for the History of Medicine and Health, 78, 1 (2021), 74–95, 83–93. The area around Białystok and Grodno was part of the projected ‘Memel-Narew region’ of the *Generalplan Ost.* See Gerlach, *op. cit.* (note 64), 113. For the *Generalplan Ost*, see Mechtild Rössler and Sabine Schleiermacher (eds), *Der ‘Generalplan Ost’: Hauptlinien der nationalsozialistischen Planungs- und Vernichtungspolitik* (Berlin: Akademie, 1993); Sabine Schleiermacher , ‘Soziobiologische Kriegführung? Der Generalplan Ost’, Berichte zur Wissenschaftsgeschichte, 19, 2–3 (1996), 145–5611630274 10.1002/bewi.19960190213; Karl-Heinz Roth , ‘Ärzte als Vernichtungsplaner: Hans Ehlich, die Amtsgruppe III B des Reichssicherheitshauptamtes und der nationalsozialistische Genozid 1939–1945’, in Michael Hubenstorf et al. (eds), Medizingeschichte und Gesellschaftskritik: Festschrift für Gerhard Baader (Husum: Matthiesen, 1997), 398–419 11619558; Isabel Heinemann , ‘Wissenschaft und Homogenisierungsplanungen für Osteuropa: Konrad Meyer, der “Generalplan Ost” und die Deutsche Forschungsgemeinschaft’, in Isabel Heinemann and Patrick Wagner (eds), Wissenschaft – Planung – Vertreibung: Neuordnungskonzepte und Umsiedlungspolitik im 20. Jahrhundert (Stuttgart: Steiner, 2006), 45–72 .

146 Weindling, *Epidemics*, *op. cit.* (note 15), 255.

147
*Ibid.*, 200–5, 225–8, 250, 256; Paul Weindling , ‘Heinz Zeiss, German Medicine and the Holocaust’, in Dorothy Porter and Roy Porter (eds), Doctors, Politics and Society: Historical Essays (Amsterdam: Brill, 1993), 174–877691463; Weindling, ‘Die weltanschaulichen Hintergründe’, *op. cit.* (note 15); Bruns, *op. cit.* (note 115), 154–8; Allen, *op. cit.* (note 15), 89–92, 120; Schütz, *op. cit.* (note 16), 166–7. For Heinrich Zeiss’s (1888–1949) geo-medicine paradigm, see also Sabine Schleiermacher , ‘Der Hygieniker Heinz Zeiss und sein Konzept der “Geomedizin des Ostraums”’, in Rüdiger vom Bruch (ed.), Die Berliner Universität in der NS-Zeit, Vol. 2 (Stuttgart: Steiner, 2005), 17–34 .

## Long descriptions

### Figure 5. Long description

In the foreground on the right, a pair of hands holds a dark glass bottle beneath a metal dispensing nozzle. A thin, dark tube connects this nozzle to a larger glass vessel held aloft by a metal ring stand in the center. Behind the dispensing area, dozens of small, dark glass vials are arranged in neat, dense rows on the laboratory bench. To the left, a wooden table holds a large dark container and a small, rounded object. The background consists of a tiled wall, and the overall lighting is harsh, creating high contrast between the dark liquids and the light-colored surfaces of the laboratory at the Hygiene Institute of the Waffen-S S.

Navigate back to Figure 5..

